# Harnessing Synergistic Cooperation of Neighboring Active Motifs in Heterogeneous Catalysts for Enhanced Catalytic Performance

**DOI:** 10.1002/adma.202501960

**Published:** 2025-05-12

**Authors:** Peng Yuan, Ching Kit Tommy Wun, Tsz Woon Benedict Lo

**Affiliations:** ^1^ State Key Laboratory of Chemical Biology and Drug Discovery Department of Applied Biology and Chemical Technology The Hong Kong Polytechnic University Hung Hom Kowloon Hong Kong 100872 China; ^2^ The Hong Kong Polytechnic University Shenzhen Research Institute The Hong Kong Polytechnic University Shenzhen 518057 China; ^3^ PolyU‐Daya Bay Technology and Innovation Research Institute The Hong Kong Polytechnic University Huizhou Guangdong 516083 China

**Keywords:** active motifs, electronic structures, geometric distances, heterogeneous catalysts, synergistic cooperation, transition metals

## Abstract

Understanding the intricate interplay between catalytically active motifs in heterogeneous catalysts has long posed a significant challenge in the design of highly active and selective reactions. Drawing inspiration from biological enzymes and homogeneous catalysts, the synergistic cooperation between neighboring active motifs has emerged as a crucial factor in achieving effective catalysis. This synergistic control is often observed in natural enzymes and homogeneous systems through ligand coordination. The synergistic interaction is especially vital in reactions involving tandem or cascade steps, where distinct active motifs provide different functionalities to enable the co‐activation of the reaction substrate(s). Situated within a 3D spatial domain, these catalytically active motifs can shape favorable catalytic landscapes by modulating electronic and geometric characteristics, thereby stabilizing specific intermediate or transition state species in a specific catalytic reaction. In this review, we aim to explore a diverse array of the latest heterogeneous catalytic systems that capitalize on the synergistic cooperativity between neighboring active motifs. We will delve into how such synergistic interactions can be utilized to engineer more favorable catalytic landscapes, ultimately resulting in the modulation of catalytic reactivities.

## Graphical abstract

This review examines the critical role of neighboring active motifs in heterogeneous catalysts, emphasizing how their synergistic cooperativity can be rationally designed to modulate catalytic properties. The discussion centers on the modulation of electronic and geometric structures, categorizing the catalytic systems into metal‐acid (Brønsted/Lewis) pairs, metal‐base pairs, and metal‐metal pairs. The goal is to provide a systematic overview that offers insights into the design and characterization of next‐generation multifunctional catalysts.

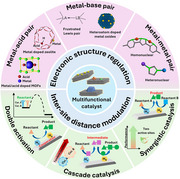

## 1 Introduction

Catalysis is a cornerstone of modern society, influencing a wide array of industries from energy production to environmental sustainability. Key chemical processes, such as the Haber‐Bosch process,^[^ ^1^ ^]^ catalytic cracking,^[^ ^2^ ^]^ and polymerization reactions,^[^ ^3^ ^]^ have substantially advanced human development. To enhance these catalytic processes, a deep understanding of the active sites within catalysts has become essential. In 1925, Hugh Stott Taylor introduced the concept of active sites–specific atoms or ensembles of atoms on a catalyst's surface that drive catalytic reactions.^[^ ^4^ ^]^ This perspective shifted the focus from the entire catalyst structure to these pivotal active motifs, revealing their critical role in facilitating reactions.^[^ ^5^ ^]^ Recent advancements in characterization techniques, such as aberration‐corrected scanning transmission electron microscopy (AC‐STEM) and synchrotron‐based methods like X‐ray absorption spectroscopy, have greatly enhanced our ability to uncover the intricate structure‐activity relationships between active sites and catalytic performance. The interplay between different binding modes of reactants and active sites can dramatically influence catalytic activity and selectivity.

In some more complex reactions, such as those involving C─C and C─O bond formations, the contribution of multiple active sites is often necessary, as the adsorption and activation of reactants and intermediate species may require the collaboration of several active motifs.^[^ ^6^ ^]^ Today, the rapid progress in heterogeneous catalysis is paving the way for the design and synthesis of multifunctional catalysts that outperform traditional models. These innovative approaches enable precise tuning of the electronic and geometric structures of neighboring active sites, harnessing their synergistic effects for enhanced catalytic performance.^[^ ^7^ ^]^ Therefore, investigating the interactions between adjacent active sites has emerged as a pivotal trend in the pursuit of high‐performance catalysts, as their cooperative behavior is vital for achieving efficient catalysis.

**Scheme 1:**
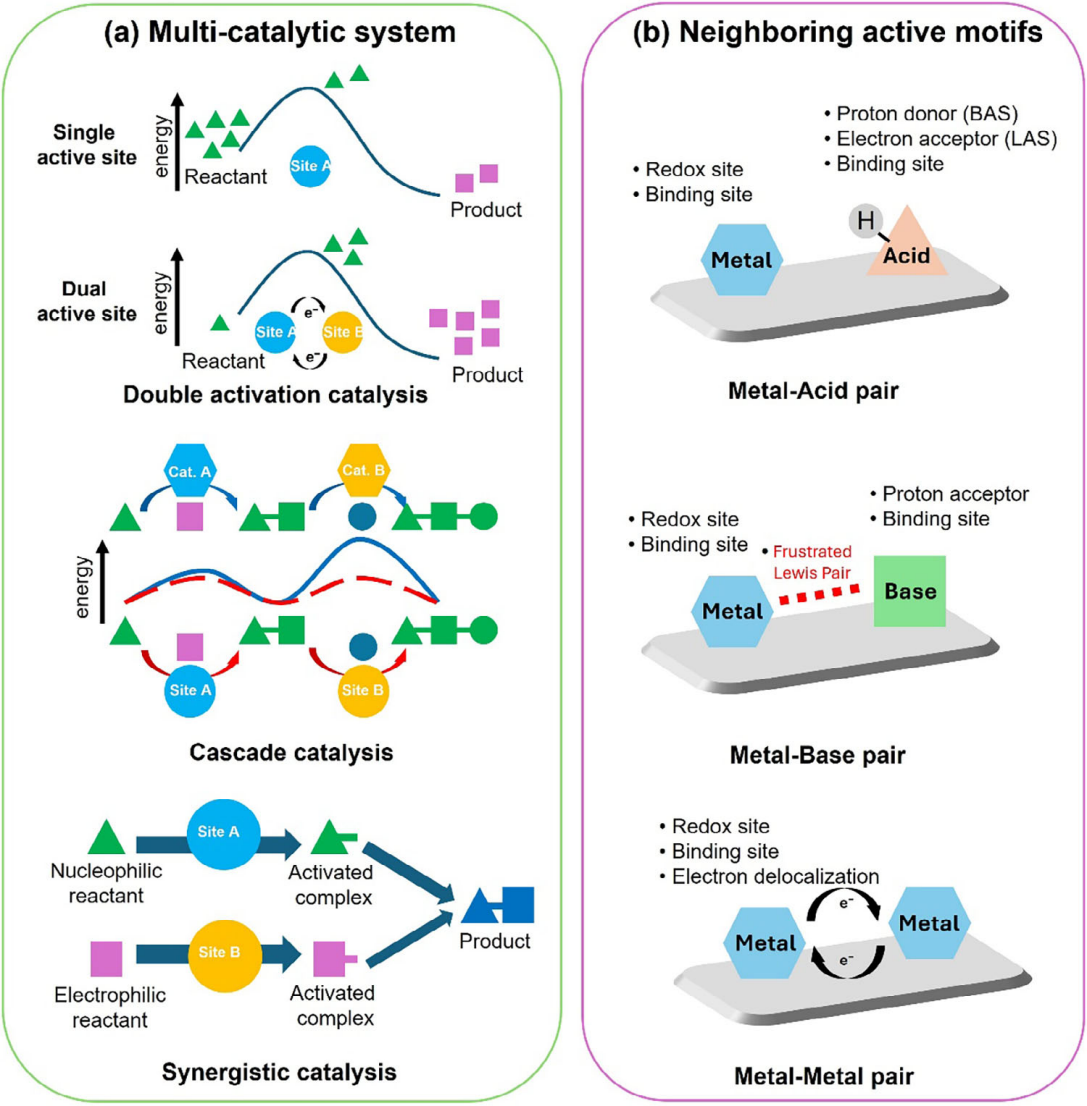
a) A schematic illustration of three major multi‐catalytic systems: double activation catalysis, cascade catalysis, and synergistic catalysis. b) A schematic illustration of the classification of active motif pairs, including metal‐acid (Brønsted/Lewis) pairs, metal‐base pairs, and metal‐metal pairs, along with their corresponding functions.

In the cutting‐edge realm of multifunctional catalysts, neighboring active motifs play a pivotal role in lowering reaction activation barriers and enhancing selectivity for target products by creating novel reaction pathways. Drawing inspiration from biological enzymes, we learned that the synergistic cooperation among neighboring active motifs is essential for achieving effective catalysis.^[^ ^8^, ^9^, ^10^ ^]^ Although the diversity of metal atoms in the active sites plays a significant role in shaping the catalytic landscape, the tertiary domain—akin to protein scaffolds—highlights the crucial importance of a confined environment. Such configurations not only enhance adsorption but also stabilize specific transition states and reaction intermediates, driving catalytic efficiency. This synergistic effect, born from the integration of multiple active sites, fosters improved orbital interactions between metals and adsorbates, leading to more effective orbital overlaps. By optimizing σ or π donation and π* back‐donation, these motifs can enhance the activation of adsorbates, thereby boosting catalytic efficiency.^[^ ^11^, ^12^ ^]^ This synergy not only sheds light on complex catalytic mechanisms but also enables the tailored design of catalysts, paving the way for advanced multi‐catalytic systems suited for diverse applications. To tackle the activation of various substrates, researchers have developed three primary types of multi‐catalytic systems (**Scheme** **1**a): double activation catalysis, cascade catalysis, and synergistic catalysis.^[^ ^13^ ^]^

In double activation catalysis, two catalytic sites collaborate to activate a single reactant through two main pathways: one active motif adsorbs the substrate while the other facilitates activation via electron transfer,^[^ ^14^ ^]^ or through a cascade electron transfer between the active motifs and the substrates.^[^ ^15^ ^]^ This strategy has proven effective in the synergistic activation of nitrogen (N₂), where hard acid metal sites with high ionization potential accept electrons from N₂, while soft acid sites donate electrons, culminating in efficient ammonia formation.^[^ ^15^ ^]^

Similarly, cascade catalysis involves the successive activation of a single reactant across multiple catalytic sites, where the reactant's role may oscillate between nucleophile and electrophile, or follow distinct reaction pathways at each step.^[^ ^16^, ^17^ ^]^ For instance, in the electrocatalytic reduction of CO₂ into multicarbon (C_2+_) products, cascade electrocatalysts like Ag─Cu first reduce CO₂ at Ag sites to generate CO species, which then spill over to Cu sites for subsequent *CO protonation and asymmetric C─C coupling to valuable C_2+_ products.

Last, synergistic catalysis encompasses multiple reactants, including both nucleophiles and electrophiles, necessitating various active motifs to drive the reaction. This approach allows for the coupling of different reactants and stabilizes the transition state or reaction intermediates throughout the reaction.^[^ ^18^, ^19^ ^]^ In the hydrogenation of unsaturated chemical bonds, one catalytic site activates H₂ through homolytic or heterolytic dissociation, while another site adsorbs and activates the unsaturated bond.^[^ ^18^ ^]^ The cooperative action of these catalytic sites ultimately dictates both efficiency and selectivity, marking a significant advancement in catalytic science.

Among the various active motifs, transition metals (TMs) stand out as crucial components in the creation of multifunctional catalysts. Their unique *d*‐orbital characteristics confer exceptional catalytic performance, enabling variable oxidation states, facilitating complex formation, and exhibiting remarkable redox properties.^[^ ^20^ ^]^ The electronegativity of transition metals is closely related to their functional differentiation in catalytic reactions. For high‐valent metal species (e.g., Zn^2^⁺, Al^3^⁺), their high electronegativity enhances the attraction of their empty orbitals toward electron pairs, enabling them to act as strong Lewis acid sites (LASs) in reactions (e.g., Al^3^⁺ accepting π‐electrons via empty orbitals in Friedel‐Crafts alkylation).^[^ ^21^, ^22^ ^]^ In contrast, metals with variable valence states (e.g., Fe^3^⁺/Fe^2^⁺, Co^3^⁺/Co^2^⁺) exhibit lower electronegativity, which reduces the energy barrier for *d*‐orbital electron transfer, facilitating electron exchange with substrates through redox cycles (e.g., Cu^2^⁺/Cu⁺ promoting •OH radical generation in Fenton reactions).^[^ ^23^, ^24^, ^25^ ^]^ In summary, the electronegativity of metals governs their role differentiation and performance optimization in catalytic systems by regulating electronic structures and orbital interactions. This review delves into TM‐based heterogeneous catalysts developed over the past five years, focusing on the design and production of multifunctional catalysts that feature diverse active motif pairs (Scheme 1b), including metal‐acid (Brønsted/Lewis) pairs, metal‐base pairs, and metal‐metal pairs.

We aim to offer an extensive understanding of the electronic structure of these catalysts and the precise spatial arrangement of active motifs at the atomic level through coordination chemistry. Central to this discussion is the shaping of “a favorable catalytic landscape”, where we tend to define it as the tailored atomic and electronic structures of catalysts for the stabilization of intermediate/transition state species that favor specific catalytic reaction pathways through synergistic electronic and geometric effects. Our work emphasizes that optimal spatial arrangements are reaction‐specific rather than universal, as evidenced by the varying synergistic distances required for different catalytic processes. Additionally, the preparation and characterization of neighboring active sites with atomically precise structures—key descriptors of the catalytic landscapes—are central to our exploration. By thoroughly investigating these active sites, we aspire to gain deeper insights into the nature of catalytic reactions, laying a robust theoretical foundation for the optimization of catalyst performance. Finally, on the basis of current research advancements, we will also highlight future research directions. We hope that these insights will inspire innovative catalyst design, accelerate the commercialization of efficient heterogeneous catalysts, and make significant contributions to related fields.

## 2 Design of Neighboring Active Motifs

**Scheme 2:**
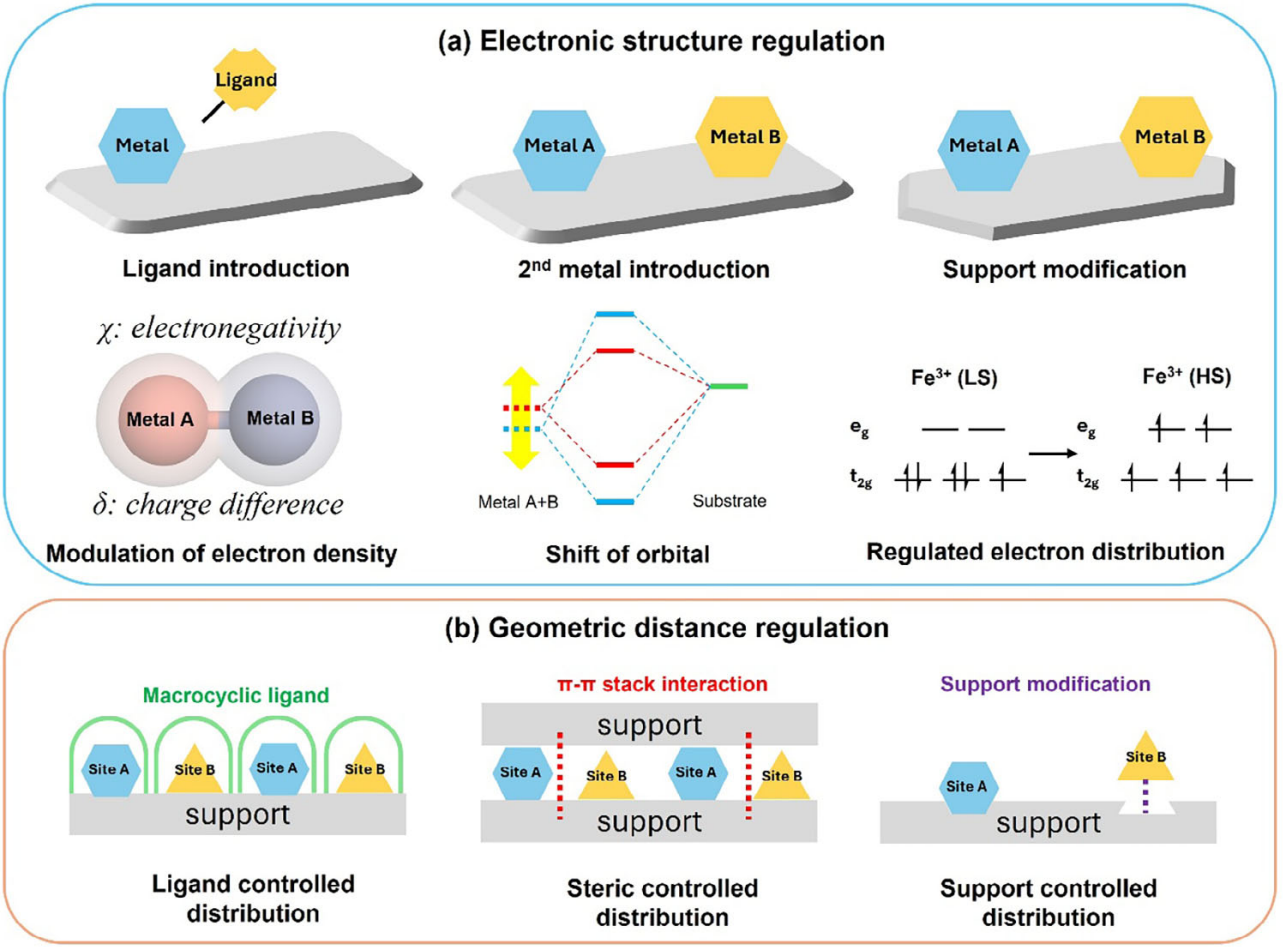
A schematic illustration of strategies for a) modulating neighboring active motifs, including the introduction of ligands and a second metal, and support modification for electronic structure regulation, and b) geometric distance regulation by addition of macrocyclic ligand, confinement by 2D support layer, and precise engineering of active sites within porous crystalline support.

A comprehensive understanding of the coordination chemistry of neighboring active motifs and the characteristics of the host matrix is crucial for designing highly effective metal‐based multifunctional catalysts, paving the way for broader industrial applications. Precise regulation of the electronic structure and geometric distance between neighboring active sites—such as metal‐acid, metal‐base, and metal‐metal pairs—plays a vital role in determining catalytic performance. TM active sites often feature empty or partially filled *d*‐orbitals, allowing them to coordinate with non‐metal heteroatoms (C, N, S, P, and O) from ligand molecules or binding sites on solid supports (e.g., metal oxides, MOFs, zeolites, carbon‐based materials, etc.). Modulating electronic structures encompasses the degree of *d*‐band filling, spin state, charge difference, and electronegativity (**Scheme** **2**a).^[^ ^11^, ^12^ ^]^ This modulation is primarily achieved through coordination engineering involving doped heteroatoms and host matrix modifications, including point vacancy engineering. The interfacial electronic coupling between isolated metal atoms and solid substrates facilitates efficient charge transfer, thereby enhancing catalytic performance. By tailoring the substrate materials, it is possible to optimize both the density of metal‐support interaction sites and the redox characteristics, which are crucial for catalytic efficiency. Additionally, the spacing between neighboring active motifs can be adjusted to achieve synergistic cooperation (Scheme 2b). When the spacing between these motifs decreases to a critical threshold, their interactions can synergistically modulate the electronic structure of the active sites, notably improving catalytic efficiency.

### 2.1 Modulation in Catalytic Chemistry through Electronic Structure Regulation

**Figure 1:**
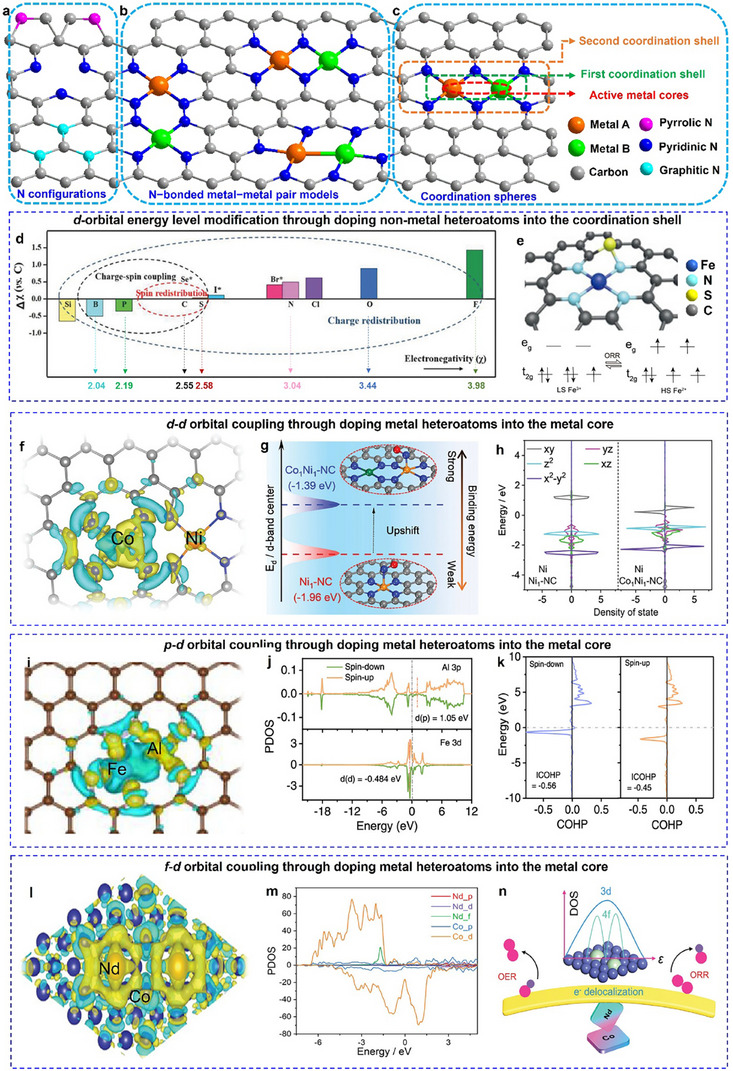
Regulating the electronic structure of the active sites by introducing secondary heteroatoms into the coordination spheres. a) Different N configurations (pyridinic, pyrrolic, and graphitic N) in metal‐based catalysts. b) Three pyridinic N‐bonded metal‐metal pair models. c) Coordination spheres of metal‐based catalysts with metal‐metal pairs. d) Classification of the origin of doping effects as charge‐redistribution, spin redistribution, and charge–spin coupling based on the relative electronegativity of heteroatom dopants. Se, Br, and I are labeled with the superscript of *, denoting that these heteroatoms are too large to be incorporated into the carbon lattice, and spatial distortion has to be considered as an important factor for inducing catalytic activities rather than pure contributions from charge and spin redistributions. Reproduced with permission.^[^ ^32^ ^]^ Copyright 2018, WILEY‐VCH Verlag GmbH & Co. KGaA, Weinheim. e) Iron spin‐state tuning in S‐doped Fe‐N‐C single‐atom catalysts enhances ORR activity. Reproduced with permission.^[^ ^33^ ^]^ Copyright 2021, Wiley‐VCH GmbH. f) Charge density difference of Co_1_Ni_1_‐NC, with the yellow and blue colors indicating regions of electron accumulation and depletion, respectively. g) Schematic depiction of the positive displacement of the *d*‐band center arising from the synergistic interaction between metallic atoms. h) Partial density of states (PDOS) of Ni *3d* for Ni_1_‐NC and Co_1_Ni_1_‐NC. f–h) Reproduced with permission.^[^ ^34^ ^]^ Copyright 2024, Wiley‐VCH GmbH. i) Charge density difference of FeN_3_‐O‐AlN_3_. j) PDOS for Al 3*p* orbit and Fe‐3*d* orbit of FeN_3_‐O‐AlN_3_. The red dotted lines denote the *d*‐band centers, while the gray dotted line denotes the Fermi level. k) The COHP for Al─Fe bond in FeN_3_‐O‐AlN_3_. Reproduced with permission.^[^ ^35^ ^]^ Copyright 2024, American Chemical Society. l) Charge density difference of Nd/Co@NC. m) PDOS for Nd/Co@NC. n) Schematic illustration of the essential role of Nd in balancing OER and ORR activity. Reproduced with permission.^[^ ^36^ ^]^ Copyright 2022, Wiley‐VCH GmbH.

The electronic structure of active sites can be optimized through strategies such as introducing secondary heteroatoms into the coordination spheres (coordination shells and active metal cores) (**Figure** **1**c) or engineering defects in support materials. These doping modifications provide strong anchoring for foreign metal atoms, ensuring the stability of atomically dispersed catalytic sites. Moreover, doping can modulate the charge density of the catalyst, alter the energy barriers for intermediate reactions, and hence the overall catalytic activity.

#### 2.1.1 Introducing Secondary Heteroatoms into Coordination Spheres

The coordination spheres of heterogeneous catalysts consist of coordination shells (first and second coordination shells) and active metal cores. In the first coordination shell, metal centers directly coordinate with non‐metal heteroatoms like C, N, S, P, and O. Nitrogen atoms are often doped directly into carbon matrices due to their higher electronegativity (*χ* = 3.04) and similar atomic size to carbon (*χ* = 2.55). We will discuss this sub‐section using N‐doped carbon materials (NCs) as they can finely modulate the electronic structures in a structured manner. NCs are gaining popularity for their uniformly distributed pore/defect structures, which effectively capture incoming metal sites through *sp^2^*‐bonding with lone pairs at the edges of pores. Different configurations of M−N sites (pyridinic, pyrrolic, and graphitic N) in metal‐based catalysts are closely linked to their unique electronic structures (Figure 1a). For instance, graphitic N acts as an electron donor due to its high‐energy π* electron, while pyridinic N serves as an electron acceptor because of its π‐electron deficiency and lone pair, imparting Lewis basicity. Pyrrolic N, with *sp^3^* hybridization, contributes electrons to the π‐system but is thermally unstable, often converting to graphitic N at elevated temperatures.^[^ ^26^ ^]^ Extensive research on pyridinic N‐bonded carbon materials has led to the proposal of three pyridinic N‐bonded metal‐metal pair models: N₂─M_A_─N₂─M_B_─N₂, N₃─M_A_─M_B_─N₃, and N₂─M_A_─N₄─M_B_─N₂ (Figure 1b). The N₃─M_A_─M_B_─N₃ model exhibits noticeably higher binding energies (0.82 ─ 2.97 eV higher than N₂─M_A_─N₂─M_B_─N₂ and 0.68 ─ 2.30 eV higher than N₂─M_A_─N₄─M_B_─N₂) due to the strong interaction of unsaturated dangling N atoms with the metal centers.^[^ ^27^ ^]^

Doping with relatively less electronegative P (*χ* = 2.19) allows for modification of the electronic structure of active metal sites through asymmetric coordination, enabling electron donation to active metal atoms. Conversely, doping with more electronegative S (*χ* = 2.58) and O atoms (*χ* = 3.44) would withdraw electron density from the metal sites (Figure 1d). Leveraging their differing electronegativities, doped‐O motifs in NC matrices facilitate the selective coordination for N with Fe and O with Co. This approach effectively addresses the challenge of random metal‐metal pair combinations, resulting in the formation of an N_2_─Fe─NO─Co─O_2_ configuration with bridged N and O atoms. The strong coupling between Fe─N_3_ and Co─O_3_ modifies the *d*‐orbital energy levels of the metal atoms, resulting in electron redistribution within the Co‐Fe pairs. The lower occupancy of the Fe 3*d*_z_^2^ orbital enhances electron transfer from O to the Fe sites, while the higher Co 3*d*_z_^2^ occupancy reduces transfer energy due to the lower electron affinity of the Co sites. Together, these factors can collectively impact catalytic activity.^[^ ^28^ ^]^ Similarly, the N‐doped graphene nanosheets (CNG) can also stabilize the Fe‐Ni dual‐site by forming N_2_─Ni─O_2_─Fe─N_2_ moieties through structure reconstruction. These moieties are proposed to be the “actual” active species responsible for oxygen evolution reaction (OER) when using this catalyst. The density functional theory (DFT) simulations suggest that the OER pathway of NiFe‐CNG follows a dual‐site mechanism involving Ni─O─Fe moieties. Both Ni and Fe sites participate in the *OH deprotonation process, leading to bridging O_2_ formation atop the Ni─O─Fe bonds. The differing spin densities indicate that Ni adopts low‐spin configurations while Fe is high‐spin, suggesting unpaired Fe electrons in the *t*_2g_ and *e*_g_ orbitals optimize charge transport and adsorption of OER intermediates.^[^ ^29^ ^]^ By doping S atoms into NCs, a dual‐site catalyst featuring CoN_3_S and MnN_2_S_2_ configurations used in oxygen reduction reaction (ORR) has been reported. The inclusion of Mn and S alters the electron configuration symmetry of the Co 3*d* orbital, reduces the *d*‐band center of the Co site, and enhances the desorption of the OH intermediate.^[^ ^30^ ^]^ Similarly, a finely controlled P‐doping technique has been employed to synthesize Fe‐Co dual‐site on NCs. The achieved catalysts displayed N_2_─Fe─NP─Co─N_2_ configurations with lower formation energy compared to N_2_─Fe─N_2_─Co─N_2_. The doped‐P species greatly alters the electronic localization and spin polarization of active sites, resulting in an O bridging site between the Fe and P atoms and OH* on top of the P atom.^[^ ^31^ ^]^ These modifications of the first coordination shell can impact both catalytic activity and stability, as confirmed by recent studies showing the superior ORR performance of catalysts with O/P/S‐coordination compared with those relying solely on N‐coordination.

The electronic regulation of the metal center can also be influenced by other coordination shells through heteroatomic doping or functional group engineering. Compared with the first shell atoms, these modifications affect the electron density indirectly and more moderately.^[^ ^37^, ^38^ ^]^ In Ru‐N‐C catalysts, sulfur preferentially coordinates with nitrogen rather than directly with ruthenium, establishing a directional electron transfer pathway (S→N→Ru) that enhances the metal center's electron density. This electronic redistribution weakens intermediate adsorption, as confirmed by EXAFS analysis showing S‐induced Ru‐N bond elongation (1.886 Å vs undoped) from asymmetric structural distortion. Notably, direct Ru–S coordination would produce opposite electronic effects by withdrawing electron density from the active site, highlighting the critical advantage of second‐shell S doping for optimal catalytic performance.^[^ ^37^ ^]^ Besides, spatial S‐bridge ligands have been introduced to regulate the coordination environment of Fe–Co dual‐sites in NC. The neighboring Co─N_4_ active center and adjacent S ligand bridged over spatially separated graphitic layers can synergistically regulate the *d*‐orbital electronic structure of the Fe─N_4_ active center to optimize the adsorption‐desorption of oxygen intermediates, leading to a considerably reduced thermodynamic barrier for acidic ORR.^[^ ^39^ ^]^ Beyond this, incorporating S heteroatoms into the second coordination shell of M‐N‐C catalysts enables precise modulation of the metal center's spin state (e.g., stabilizing low‐spin Fe^3^⁺), optimizing the adsorption/desorption energetics of key intermediates (e.g., OH), and triggering dynamic spin transitions (LS ↔ HS) during catalytic reactions(Figure 1e).^[^ ^33^ ^]^ Besides the doped atoms, the addition of ligands is also feasible in the modulation of the electronic structure of the metal center in heterogeneous catalysts. By employing the ligand field strengths binding to the metal centers, the distribution of the electron will be affected and further influence the electron transfer between the metal center and substrate molecules. In ORR reaction, a Co‐based catalyst is an effective heterogeneous catalyst that regulates the electron transfer during the electrocatalytic ORR reaction.^[^ ^40^ ^]^ By employing the ligands with different ligand field strengths, the position of the unpaired electrons in the Co center will vary, which can be identified by XPS and XAFS analysis. According to the Co 2*p* spectra of Co catalyst binding with different ligands, we can see that the spin‐orbit splitting is narrow with the increasing ligand field strength, and the satellite peaks also decrease with the growing ligand field strength. Also, the shifting of the white line to higher eV follows the trend of increasing ligand field strength. The Co catalyst with strong field ligand also exhibits significant selectivity (94%) in ORR reactions.

In addition to the coordination shells, the electronic structure of metal centers can be effectively tuned via direct modification of the metal core composition. A key distinction emerges between homonuclear and heteronuclear systems: while identical metal pairs (e.g., Fe–Fe) exhibit constrained electronic modulation due to their uniform electronegativity, mixed‐metal systems (Fe‐Co/Ni/Zn) demonstrate enhanced functionality through intrinsic charge polarization.^[^ ^41^, ^42^, ^43^ ^]^ This effect stems from electronegativity differences (*χ*_Fe_ = 1.83, *χ*_Co_ = 1.88, *χ*_Ni_ = 1.91), which drive directional electron transfer from Fe to Ni (≈ 0.14 |e| per atom in FeNi₃ systems) and create complementary electronic states. The resulting interfacial charge gradients simultaneously enable efficient electron transfer through enriched *d*‐bands (e.g., Ni‐3*d*) while maintaining adjacent electron‐deficient sites (e.g., Fe‐3*d*) for intermediate stabilization, thereby optimizing multistep reaction pathways.^[^ ^41^ ^]^

Doping heterometallic atoms into the active metal cores can effectively modify the electronic structure of active sites through orbital hybridization between metal‐metal pairs, thereby enhancing synergistic catalytic properties. The *d*–*d* orbital coupling strategy can be utilized to create heteronuclear dual‐atom sites involving 3*d* TMs, such as Co‐Ni pairs. This strategy greatly enhances the affinity between the metal atomic sites and nitric oxide (NO), thereby improving electrocatalytic performance for NO reduction. The interaction between the Co and Ni sites results in crucial electron accumulation at the Ni sites, which raises the Ni *d*‐band center closer to the Fermi level, thereby increasing the NO adsorption capacity (Figure 1f–h). This optimized electronic configuration promotes the transfer of 3*d_xy/yz_* electrons from Ni to the 2*π* orbitals of NO, effectively weakens the N≡O triple bond and accelerates the electrooxidation of NO.^[^ ^34^ ^]^ Compared to commonly used 3*d* TMs such as Fe, Co, and Ni, 4*d* TMs (low‐spin) like Nb and Mo possess fewer *d* electrons with more unoccupied orbitals, allowing them to interact more strongly with 3*d* metal sites, further affecting the catalytic activity and structural stability of these catalysts. Inspired by this principle, an asymmetric Fe‐Nb dual‐site with NS─Fe─N₂─Nb─N₂ configuration was developed, exhibiting high ORR performance and durability. The strong interaction between Fe and Nb atoms optimizes the desorption energy of critical ORR intermediates like OH, positioning the Fe─OH adsorption energy within the optimal range on the ORR volcano plot. Furthermore, the incorporation of Nb reinforces the Fe─N bonds and reduces Fe demetalation, improving its structural stability.^[^ ^44^ ^]^ Similarly, the introduction of Mo atoms into the Fe─N₄C catalyst that forms Mo‐Fe dual‐site also modulates the electronic configuration, which downshifts the Fe *d*‐band center and weakens ORR intermediate adsorption. This tailored architecture elongates the O─O bond and promotes O─O cleavage, achieving superior ORR activity in O₂‐saturated 0.1 m HClO₄ electrolyte.^[^ ^45^ ^]^

Beyond *d*‐block metal pairs, modification using *p*‐block species offers an alternative approach due to the spherical symmetry of their *p* orbitals, which facilitates hybridization with the more complex *d* orbitals of 3*d* TMs. Since *p* orbitals generally possess higher energy levels than *d* orbitals, *p*–*d* hybridization provides a promising strategy for modulating the binding strength of intermediates, resulting in enhanced catalytic activity. For example, a heteronuclear dual‐site catalyst featuring asymmetric Fe─Sn active sites (with Fe···Sn = 2.22 Å) embedded within a 2D nitrogenated graphene nanosheet (C₂N) demonstrated remarkable ORR activity. The strong *p*‐*d* orbital hybridization between Sn and Fe promoted electron delocalization and lowered the energy barrier for *OH protonation, thereby enhancing the catalytic efficiency.^[^ ^46^ ^]^ Similarly, the introduction of a *p*‐block Al modulator induces asymmetric electron distribution through *p*–*d* orbital hybridization in Fe─Al atom pairs, optimizing oxygen intermediate desorption strength by regulating the Fe *d*‐band center and enhancing ORR activity (Figure 1i–k).^[^ ^35^ ^]^

In addition to NC matrices, the introduction of *p*‐block metal ions, such as Al^3^⁺, Ga^3^⁺, and In^3^⁺, can also affect the electronic property of metal oxides through promoted *p*–*d* orbital hybridization between the *p* orbitals of the Group 13 elements and the *d*‐orbital of the support metal motifs.^[^ ^47^ ^]^ Similarly, the *p*‐*d* hybridization strategy can be extended to *p*‐block metal‐doped Cu catalysts, through the formation of Cu–Ga, Cu–Al, and Cu–Ge pairs, to boost electrocatalytic CO_2_ reduction reaction (CO_2_RR) for C_2+_ production by enhancing the binding strength of the *CO intermediate and facilitating C−C coupling.^[^ ^48^ ^]^ Although doping with rare earth metals involves highly degenerate 4*f* orbitals, these orbitals are more localized and challenging to activate due to being shielded by 5*d* and 6*s* electrons. However, rare earth metals with unique 4*f*, 5*d*, and *6s^2^* configurations have also been shown in cases that can enhance multi‐metallic site catalytic performance through *f*–*d*–*p* orbital coupling. For instance, doping Nd into Co metal modulates the adsorption of oxygen‐containing intermediates during reversible oxygen electrocatalysis.^[^ ^36^ ^]^ This *f*‐*d* coupling improves the binding energy of these intermediates, balancing the bifunctional electrocatalytic activity of Co sites and facilitating the formation of Co─OOH intermediates through strong electronic interactions below the Fermi level (Figure 1l–n). The potential of heterometallic orbital interactions in advancing catalyst design and optimizing performance across various catalytic applications is evident from these examples.

#### 2.1.2 Manufacturing Point Defects on the Matrix Surface

In addition to introducing secondary heteroatoms into coordination spheres, the selection of solid support also plays a pivotal role in influencing the electronic structure of metal sites through metal‐support interactions (MSI). The chemical composition of the solid support greatly affects the MSI, which in turn influences charge transfer between the support and the metal center, ultimately impacting the electron density of the metal site.^[^ ^49^ ^]^ Adjusting the MSI can induce various changes in the metal site, including modifications to the electronic structure, alterations in substrate adsorption behavior, and variations in binding strength between the metal site and support. Research indicates that the oxidation state of metal active sites supported on different solid supports is closely related to the support's bandgap. This illustrates that the electronic properties of the support influence the electron density of the metal site via MSI.^[^ ^50^, ^51^ ^]^

Beyond chemical composition, the morphology of the support, particularly point defects such as cation and anion vacancies, can influence the binding sites of the support and regulate the local environment of metal active sites. The regulation of defect structures can lead to rearrangements of the atomic structure and charge distribution on the catalyst surface, thereby influencing catalytic performance.^[^ ^52^ ^]^

Carbon vacancy (V_C_) engineering on 2D carbon‐based supports plays a key role in charge distribution. This process enhances the accumulation of positively polarized charges on the active metal core, promoting the adsorption and activation of small molecules while stabilizing atomic metal or nonmetal species within catalytic centers.^[^ ^53^ ^]^ For instance, V_C_‐rich graphene‐like porous carbon embedded with Fe‐N_4_ sites has been synthesized by pyrolyzing carbon‐rich carbon nitride (C₃N₄) with trace amounts of Fe salt, demonstrating excellent performance in electrocatalytic CO₂‐to‐CO conversion with a Faradaic efficiency (FE) of 90%. Their DFT calculations indicate that Vc engineering enhances CO_2_RR performance by creating more active sites, improving electron transfer, and facilitating H₂O formation through synergistic effects with Fe─N_4_ sites and antibonding state interactions.^[^ ^54^ ^]^ Similarly, Fe‐based catalysts with V_C_ adjacent to Fe─N_4_ sites have shown remarkable efficiency in Fenton‐like reactions for phenol degradation. The presence of V_C_ results in shorter Fe─N bond lengths and higher valence states of Fe atoms, enhancing catalytic performance. Additionally, the presence of V_C_ also increases the net Bader charge of Fe atoms, allowing axial O atoms to gain electrons, which weakens Fe─O bond interactions and promotes phenol oxidation during C─O bond formation.^[^ ^55^ ^]^

**Figure 2:**
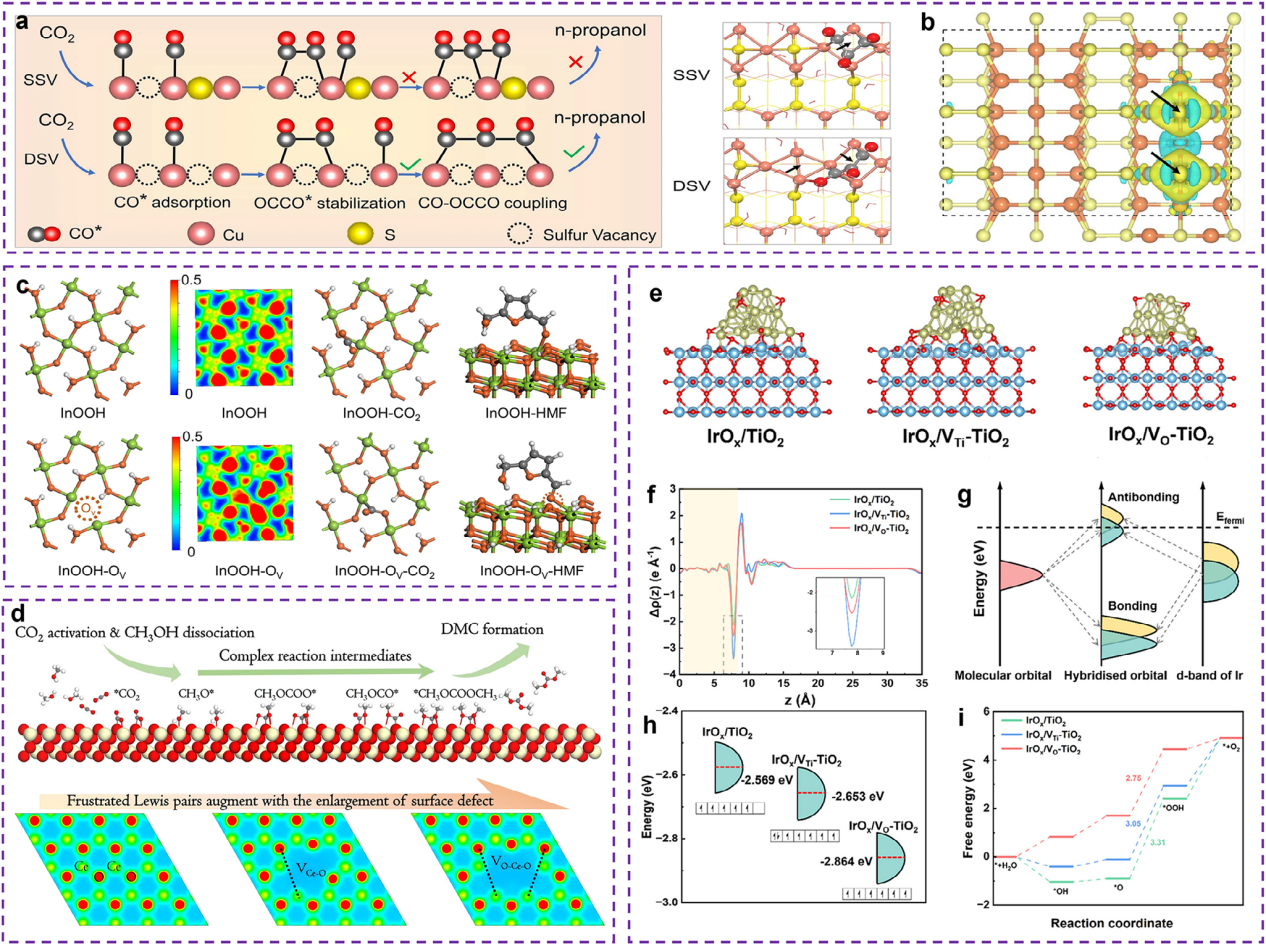
Regulating the electronic structure of the active sites by manufacturing point defects on the matrix surface. a) Mechanism of n‐propanol formation on adjacent CuS_x_‐DSV, showing the dimerization of CO─CO followed by CO─OCCO coupling. Top views of the optimized OCCOCO* intermediate configurations on (100) surface of CuS_x_‐SSV and CuS_x_‐DSV. The arrows indicate the positions of sulfur vacancies. b) The crystal structure with charge density contour plots of the adjacent double sulfur vacancy‐rich CuS_x_(100). a,b) Reproduced with permission.^[^ ^56^ ^]^ Copyright 2021, The Author(s), published by Nature Publishing Group. c) The diagrams (top view) of built models of InOOH and InOOH‐O_V_ and their corresponding ELF, and the adsorption configurations of CO_2_ and HMF. Reproduced with permission.^[^ ^59^ ^]^ Copyright 2023, The Author(s), published by Nature Publishing Group. d) Illustration of the reaction route for the direct DMC synthesis from CO_2_ and CH_3_OH on CeO_2_. Concept of FLPs constructed on a defective CeO_2_ surface and the electron‐density isosurface of pure, Ce−O diatomic vacancy, and O−Ce−O triatomic vacancy of CeO_2_, respectively, where dark dash lines label the FLPs. Theoretical calculations for IrO*_x_*/V_O_‐TiO_2_, IrO*_x_*/V_Ti_‐TiO_2_, and IrO*_x_*/TiO_2_. Reproduced with permission.^[^ ^62^ ^]^ Copyright 2022, Wiley‐VCH GmbH. (e) Optimized configurations exploited for DFT simulations. f) Planar‐integrated electron charge‐density difference. g) Schematic illustration of the influence of the *d*‐band center on the electron filling of the antibonding orbitals. h) *d*‐band center diagram and the filling of electrons. i) Gibbs free energy diagrams for the OER at *U* = 0 V. e–i) Reproduced with permission.^[^ ^61^ ^]^ Copyright 2025, American Chemical Society.

Likewise, the engineering of double‐sulfur vacancies (V_S_) has also been shown to alter electronic structures. For instance, the V_S_ in hexagonal CuS(100) planes have been found the active electrocatalytic centers for CO_2_RR through facilitated stabilization of CO* and OCCO* dimers (**Figure** **2**a). This process enables the coupling of CO and OCCO to produce the crucial C_3_ intermediate for *n*‐propanol synthesis. The synergistic effect of two adjacent V_S_ includes an increased negative charge density that allows for the adsorption of three CO species, a close distance between adjacent Cu atoms (less than 3 Å) that promotes CO─CO coupling with lower oxidation states between 0 and +2 (Figure 2b), and adequate space for relaxing the concentrated charges of OCCOCO*.^[^ ^56^ ^]^

Oxygen vacancies (V_O_) on the surfaces of certain metal oxides can create unsaturated surface sites, such as those V_O_ formed in pentacoordinate Al^3+^ on Al₂O₃^[^ ^21^ ^]^ and low‐coordinated Mg^2+^ on MgO.^[^ ^57^ ^]^ These V_O_ can regulate the electronic properties of the metal center through lattice distortion and charge redistribution on the metal oxide surface, influencing the adsorption behavior of intermediates and modulating the catalytic activities. Moreover, V_O_ can serve as anchor sites for interacting with loaded metal species, including single‐atom dispersed metal sites.^[^ ^58^ ^]^ V_O_ can also maintain metal atoms near vacancies at low oxidation states during reduction reactions, leveraging the controllability and durability of metal catalysts, such as InOOH‐V_O_ for CO_2_RR (Figure 2c).^[^ ^59^ ^]^ Similarly, Wang et al. developed Cu‐based catalysts supported by CeO₂ and ZrO₂ for the hydrogenation of CO₂ to methanol. The CO₂ conversion rates for Cu/CeO₂ and Cu/ZrO₂ at 300 °C were 15.8% and 13.2%, respectively. In contrast, CuO achieved only ≈5% conversion under similar conditions. The temperature‐programmed reduction analysis revealed that the reduction temperature of Cu species decreased from ≈350 °C (CuO) to around 270 and 300 °C for Cu/CeO₂ and Cu/ZrO₂, respectively, enhancing their reducibility for CO₂ hydrogenation. Additionally, their Raman and X‐ray photoelectron spectroscopic results demonstrated that the presence of V_O_ on CeO₂ adjusts the MSI between CeO₂ and the Cu site, promoting the formation of key carbonate intermediates during the reaction.^[^ ^60^ ^]^ Chu et al. demonstrated that sub‐2 nm IrO_x_ clusters are stabilized on TiO_2_ supports through MSI influenced by vacancy defects. The V_O_ in TiO_2_ results in the adsorbed MSI with relatively weak strength, while V_Ti_ creates the strong embedded MSI. This tunable MSI modifies the electronic structure of the IrO_x_ clusters, with a downshifted *d*‐band center caused by V_O_, which consequently lowers the energy barrier for the rate‐determining step in the OER (Figure 2e–i).^[^ ^61^ ^]^

Metal oxides with surface defects are proposed to exhibit solid frustrated Lewis pairs (FLPs) properties, consisting of surface oxygens and metal atoms near the vacancies. They can act as Lewis basic sites (LBSs) and LASs, respectively. In the study conducted by Li et al., it was observed that H_2_ activation was facilitated through a heterolytic pathway, thereby boosting the hydrogenation activity at lower temperatures. They also demonstrated that controlling vacancy clusters on the surface of CeO_2_ facilitates the construction of solid FLPs, where the sterically hindered lattice oxygen (LBSs) and Ce^3+^ (LASs) are located near the surface defects (Figure 2d). These FLPs‐based catalysts greatly enhance CO_2_ conversion to dimethyl carbonate (DMC) by lowering the barrier of the rate‐determining step (CH_3_OCOO* → CH_3_OCO* + *O).^[^ ^62^ ^]^

Recently, Wu et al. developed a pentacoordinated Al^3+^‐enriched Al_2_O_3_ through the simple calcination of a carboxylate‐containing Al precursor, which possesses abundant heterogeneous FLPs that readily promote the heterolytic activation of H_2_, promoting hydrogenation catalysis. This heterolytic activation results in the formation of both O─H^δ+^ and Al─H^δ−^ on a defective‐Al_2_O_3_ surface, contributing to the hydrogenation of polar unsaturated bonds in aldehydes or ketones.^[^ ^63^ ^]^ Chen et al. constructed high‐density FLP sites on Nb_2_O_5_ composed of low‐valence Nb (LAS) and Nb−OH (LBS) that are spatially separated by vacancies (≈4.556 Å), utilizing a thermal‐reduction promoted phase‐transition strategy. This approach preserves Nb−OH while producing abundant vacancies, resulting in a high FLP concentration. The synergy between the LASs and LBSs was shown to promote C−H stretching, achieving a high methane conversion rate of 1456 µmol g^−1^ h^−1^ for the non‐oxidative methane conversion reaction under light irradiation.^[^ ^64^ ^]^

Cation vacancies (V_M_) on the support surface can also tune the geometric surface reconstruction of metal oxides and the electronic structures of loaded single metal sites, such as NiFe double‐layer hydroxides (LDHs). As voltage increases during the OER process, the cation vacancies in NiFe‐LDH transition from simple V_M_ to V_MOH_, and finally to the reactive V_MOH‐H_. Concurrently, the catalyst surface evolves from crystalline Ni(OH)_x_ to disordered Ni(OH)_x_, eventually forming local NiOOH species. These changes highlight the critical relationship between voltage and structural evolution, ultimately influencing the catalytic performance of the precatalyst.^[^ ^65^ ^]^ Additionally, a series of Ru single‐atom catalysts (SACs) with tunable geometric and electronic structures were developed using LDHs with different cation vacancies (M^II^ or M^III^) as supports. Vacancy regulation effectively tailors the Ru−O coordination environments and electronic configurations, resulting in a higher oxidation state and fewer *d*‐state electrons for Ru. Detailed spectroscopic characterizations and theoretical calculations revealed that isolated Ru atoms anchored by M^III^ vacancies with Ru−O−Ni coordination environments play a pivotal role in promoting enhanced catalytic performance. Specifically, the M^III^ vacancies facilitate more electrons (1.50 e) transferred from Ru to the support than vacancies M^II^ (1.45 e), thus leading to higher efficiency of benzyl alcohol oxidation with a superior turnover frequency of 1331 h^−1^.^[^ ^66^ ^]^

These advancements underscore the potential of defect engineering to generate more active sites, promote faster electron transfer, and enhance the intrinsic activity of defect sites, further advancing the design of high‐performance catalysts for diverse applications.

### 2.2 Modulation in Catalysis Chemistry through Geometric Distance Regulation

Optimizing the geometric distance between neighboring active sites for key species in heterogeneous catalysis presents a significant challenge. When the active sites are too close together, adverse interactions can lead to blockage or deactivation, while excessive spacing may hinder essential synergistic effects. Achieving an optimal spatial arrangement necessitates precise synthesis and careful consideration of site coordination changes during reactions. The distance‐dependent synergistic effects in dual‐site catalysis vary according to the type of reaction. Exploring the structure‐activity relationship of dual‐site catalysts offers valuable insights for designing catalysts strategically. This understanding enables the identification of an optimal spacing that can substantially improve catalytic performance.

In brief, we will introduce three popular strategies in catalyst design that have been proven to effectively alter the inter‐site separation between the active motif sites. (1) Utilizing metal‐coordinated macrocyclic precursors ensures controlled spacing between active sites, preventing aggregation and enhancing stability. (2) Leveraging π‐π stacking interactions between 2D materials helps fabricate axial metal‐metal pairs, maintaining the distance between active sites and improving catalyst performance. (3) Support modification allows for the design of distance‐controlled active sites, optimizing the characteristics of the support material to ensure stability and efficiency during reactions. Together, these strategies effectively control active site spacing, mitigate aggregation, and enhance the overall performance of advanced dual‐site catalysts including metal‐acid (Brønsted/Lewis) pairs, metal‐base pairs, and metal‐metal pairs (also often called dual‐atom catalysts, abbreviated as DACs) in various applications.

#### 2.2.1 The Distance‐Dependent Synergistic Effects in Dual‐Site Catalysis

The distance‐dependent synergistic effects in dual‐site catalysis represent a fundamental principle governing catalytic performance across diverse reaction systems. This phenomenon manifests through precise spatial control of active sites at multiple length scales, creating optimal electronic and molecular interactions that significantly enhance reaction pathways.

**Figure 3:**
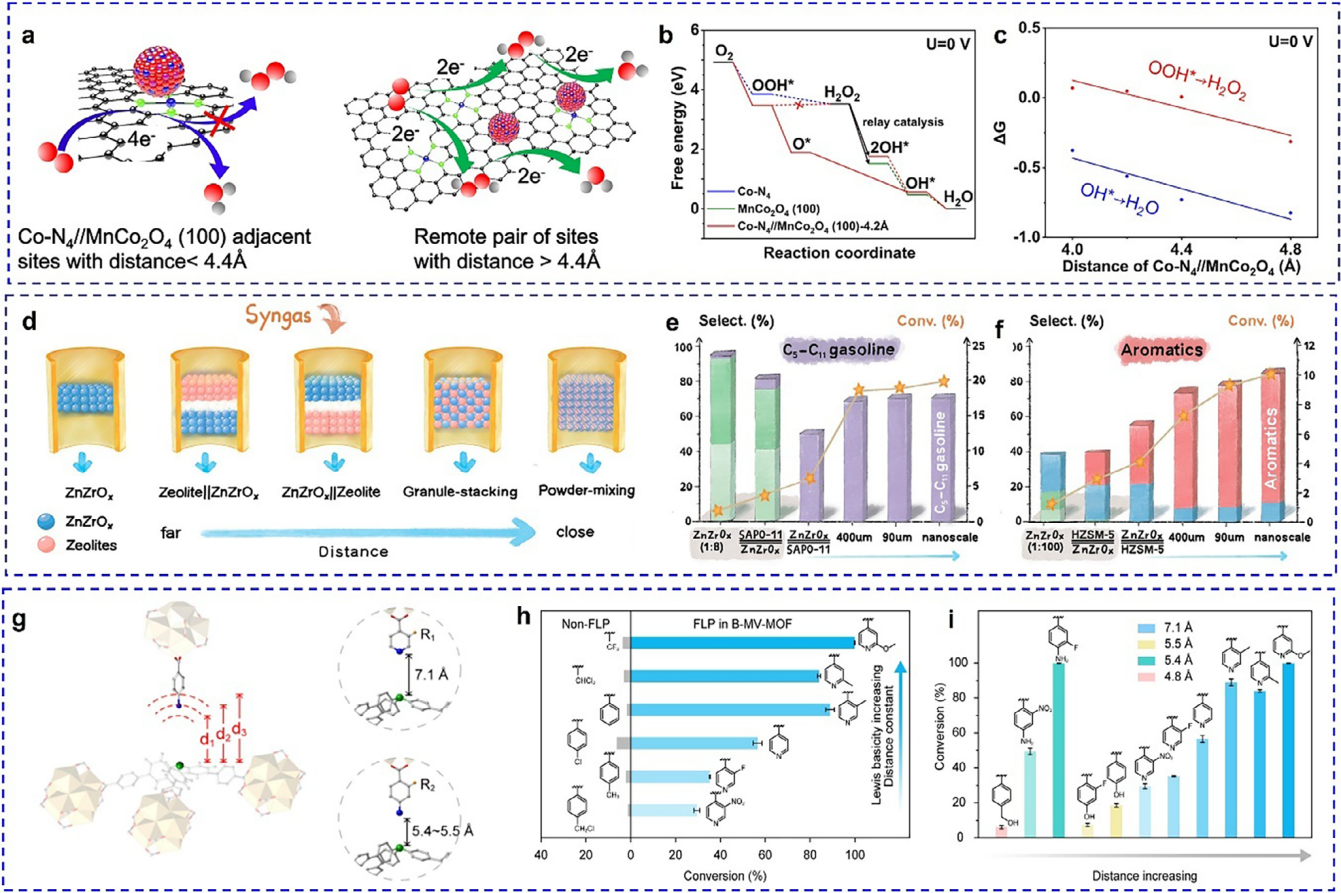
The distance‐dependent synergistic effects in dual‐site catalysis. a) Schematic diagram of the distance‐dependent synergy for the ORR on the hybrid catalyst of MnCo_2_O_4_/Co‐NC. b) Diagrams of the ORR Gibbs free energy on the indicated active sites at 0 V. c) Calculated catalytic activity plots for the production of H_2_O and H_2_O_2_ via the ORR at 0 V. a–c) Reproduced with permission.^[^ ^68^ ^]^ Copyright 2024, American Chemical Society. d) Integration manners from left to right: ZnZrO_x_; dual‐bed zeolite∥ZnZrO_x_; dual‐bed ZnZrO_x_∥zeolite; ZnZrOx + zeolite with an approximate distance of 400 or 90 µm, respectively, by stacking individual granules with different sizes; and ZnZrO_x_/zeolite prepared by the powder‐mixing method. Catalytic performance of e) STG reaction and f) STA reaction by a series of zeolite catalysts. d–f) Reproduced with permission.^[^ ^70^ ^]^ Copyright 2022, American Chemical Society. g) The design and synthesis of FLP with different fixed distances between LA and LB in MV‐MOFs. h) Comparison of the conversion efficiency between non‐FLP and FLP formed by geometry restriction in B‐MV‐MOFs. i) Assessment of catalytic performance by different FLPs in B‐MV‐MOFs. g–i) Reproduced with permission.^[^ ^71^ ^]^ Copyright 2024, American Chemical Society.

Electrochemical systems reveal even more nuanced distance effects, where the interplay between electronic coupling and molecular orientation dictates reaction mechanisms. In Fe─N₄ catalysts, the inter‐site distance critically influences ORR activity. Increasing Fe atom loading is shown to adjust the distance between the Fe─N₄ motifs, with strong interactions emerging when the spacing drops below ≈1.2 nm. These interactions alter electronic structures by modulating the Fe site spin states, enhancing intrinsic ORR activity. The effect peaks when distances approach ≈0.7 nm, beyond which activity slightly declines, highlighting the nuanced role of inter‐site spacing in optimizing neighboring active motifs.^[^ ^67^ ^]^ A MnCo₂O₄/Co‐NC hybrid electrocatalyst further exemplifies how angstrom‐scale precision controls reaction pathways in ORR. When the distance between active sites is less than 4.4 Å, the protonation of OOH* becomes energetically unfavorable, suppressing H₂O₂ formation and enabling a direct 4e⁻ ORR pathway (**Figure** **3**a–c). In contrast, distant sites support a 2e⁻ + 2e⁻ relay mechanism. This interplay illustrates how fine‐tuning active site arrangements can control reaction pathways.^[^ ^68^ ^]^ Further research on Ir–Co pair catalysts demonstrated the importance of spacing in optimizing solid‐liquid interface behavior. In situ extended X‐ray absorption fine structure (EXAFS) and synchrotron radiation infrared (SR‐IR) analyses revealed that a 7.9 Å separation balances interfacial hydroxyl generation and consumption, maximizing catalytic efficiency.^[^ ^69^ ^]^

Thermocatalytic transformations also exhibit exquisite sensitivity to active site positioning. A photo‐induced neighbor‐deposition method has also been employed to fabricate Ir_1_─Pd_1_ dual‐atom catalysts (DACs) on In₂O₃, achieving precise site‐pair spacing (≈0.25 nm). The Ir site preferentially adsorbs CO₂, while the Pd site excels in H₂ dissociation, facilitating the hydrogenation of CO_2_. Angstrom‐scale proximity reduces *H spillover and minimizes electron transfer between Pd and Ir, enhancing catalytic performance. The Ir₁Pd₁‐In₂O₃ catalyst predominantly follows a formate pathway (*HCOO) due to lower energy barriers, whereas longer inter‐site distances weaken synergy, shifting the reaction toward reverse water‐gas shift (RWGS) with increased CO production.^[^ ^72^ ^]^ Additionally, a study to unravel the proximity influence between the metal‐acid pairs was conducted by preparing a series of catalysts composed of ZnZrO_x_ and zeolite with different proximity.^[^ ^70^ ^]^ By employing different techniques to integrate two functional components, the proximity of two components varies from millimeter scale to nanoscale (Figure 3d). With the assistance of the probe reactions, the proximity requirement of the metal‐acid pairs was identified from 400 µm to the nanoscale for effective tandem reactions (Figure 3e,f).

Atomic‐level precise distance control will undoubtedly become a research hotspot in catalysis. Crystalline porous materials, particularly metal‐ and covalent‐organic frameworks (MOFs and COFs), provide ideal platforms for systematically investigating such distance‐dependent synergistic effects due to their well‐defined structures. This can be exemplified in the case of CO₂ adsorption. Studies show that maintaining adjacent metal sites at specific distances—6.3 Å for Co₂^[^ ^73^ ^]^ and 6.2 Å for Mg₂^[^ ^74^ ^]^—optimizes CO₂ binding in a linear configuration. Similarly, neighboring Cu sites are positioned within ≈4 Å, enabling dynamic Cu∙∙∙Cu interaction, which fosters an adaptive coordination mechanism that further promotes synergistic bridging pathways. This behavior is especially effective for C─C and C─X cross‐coupling reactions (X = N, O, S), characterized by low activation barriers.^[^ ^75^ ^]^ The importance of angstrom‐scale precision is further highlighted in frustrated Lewis pair (FLP) systems. By modifying the structure of some MOF‐based catalysts, the precise distance control of the FLP can be achieved with the fixed distances of 7.1, 5.5, 5.4, and 4.8 Å, respectively (Figure 3g).^[^ ^71^ ^]^ According to the catalytic conversion of cyclic carbonate from CO_2_ reaction with different FLP (Figure 3h,i), the coordination between electronic structure and the distance control of the FLP is necessary to construct an effective FLP catalyst.

Overall, these findings highlight the critical role of inter‐site distance in catalysis, demonstrating that spatial arrangement can greatly influence reaction pathways, energy barriers, and overall efficiency. This understanding provides a strategic foundation for designing advanced catalytic systems with precise control over active site positioning.

#### 2.2.2 Utilizing Metal‐Coordinated Macrocyclic Precursors

Isolated metal ions are typically employed as precursors in the synthesis of metal‐based catalysts; however, high‐temperature treatments can accelerate the random migration of these ions, resulting in their aggregation into metal nanoparticles. A promising strategy to mitigate this issue involves the use of dual‐function ligands that stabilize individual atomic sites during synthesis. These macrocyclic ligands, especially those containing N, P, and O, offer stable coordination sites for metal ions to play a crucial role in controlling the spatial arrangement of adjacent metal sites. Furthermore, the significant steric hindrance that they introduce effectively inhibits the aggregation of metal active sites during high‐temperature processes like calcination or reduction, thereby promoting the formation of atomically dispersed dual‐sites. The utilization of metal‐coordinated N‐macrocyclic rings (M─N macrocyclic complexes) has been widely validated in various studies, showcasing their capability to regulate the spatial distance between neighboring active sites. Experimental results indicated that the chelate structure of M─N_x_ remains intact during heat treatment, provided the temperature is not excessively high, allowing some stable reaction sites to persist in the material, including metal centers on carbon surfaces.^[^ ^76^ ^]^

**Figure 4:**
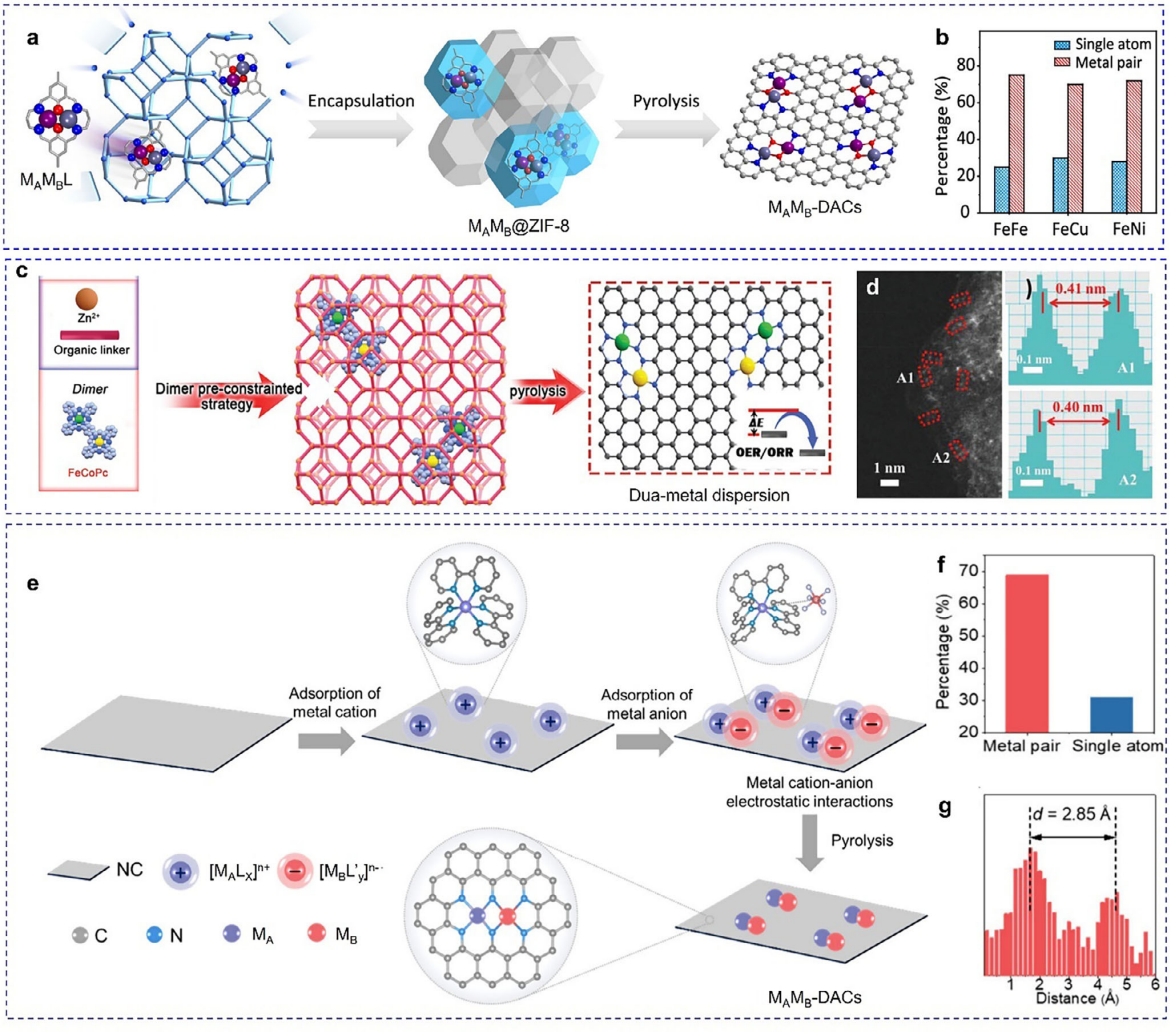
Precisely regulation of the inter‐site distance between neighboring active sites through bulky ligands. a,b) The macrocyclic precursor‐mediated approach to synthesizing a variety of DACs. Reproduced with permission.^[^ ^77^ ^]^ Copyright 2023, American Chemical Society. c,d) The pre‐constrained metal twins strategy to synthesize a variety of DACs. Reproduced with permission.^[^ ^79^ ^]^ Copyright 2021, Wiley‐VCH GmbH. e–g) The metal‐ion recognition technique to synthesize a variety of DACs. Reproduced with permission.^[^ ^81^ ^]^ Copyright 2024, American Chemical Society.

Zhang et al. presented a versatile approach using macrocyclic precursors (M_A_M_B_L) to synthesize a range of DACs, including Fe₂, Co₂, Ni₂, Fe‐Cu, and Fe‐Ni, characterized by N₂─M_A_─O₂─M_B_─N₂ motifs (**Figure** **4**a,b). The positively charged binuclear metal complexes in this method effectively reduce molecular aggregation in solution, thereby preventing the formation of clusters and nanoparticles. Additionally, Robson‐type macrocyclic ligands serve as planar coordination platforms that stabilize dual‐site species during pyrolysis, particularly when incorporated into ZIF‐8‐derived porous carbon supports. The coordination configurations were further examined using EXAFS, revealing predominant M─N/O and M─O─M interactions that resemble the M_A_M_B_L precursor. Insights into the thermal degradation pathway of FeCuL indicated that the coordination environment around the metal center is largely preserved during thermal treatment, particularly when the precursor is incorporated into a porous material. Furthermore, 70% of the metal‐metal pairs contribute to an unconventional reaction pathway during oxygen reduction (Figure 4b), primarily converting the byproduct H₂O₂ into H₂O instead of reactive oxygen species.^[^ ^77^ ^]^

Fluoro (−F) and hydroxyl (−OH) functionalized metal phthalocyanine precursors have also been used to achieve controlled synthesis of phthalocyanine networks with heteronuclear bimetallic pairs. These bimetallic phthalocyanine precursors can be in situ encapsulated into ZIF‐8 pores, followed by high‐temperature pyrolysis, leading to the formation of nearly all metal‐metal pairs. The resulting Cu‐Ni catalyst exhibited an N₂−Cu−N₂−Ni−N₂ configuration with a 0.36 nm distance between Cu and Ni, indicating the absence of direct Cu−Ni bonds, resulting in exceptional activity and stability as a CO_2_RR catalyst.^[^ ^78^ ^]^ Pre‐constrained bimetallic phthalocyanines were used to synthesize Fe‐Co DACs embedded in NC (Figure 4c), exhibiting high bifunctional activity for OER and ORR. The Fe‐Co phthalocyanine dimers maintained a locked metal ion distance and were uniformly implanted into ZIF‐8, followed by high‐temperature annealing to form N₂−Fe−N₄−Co−N₂ active motifs (Figure 4c,d). Retaining the Fe^3^⁺ and Co^2^⁺ oxidation states, these Fe–Co pairs synergistically modulated the free energy of oxygen intermediates, enhancing catalytic performance as confirmed by theoretical calculations.^[^ ^79^ ^]^ Xu et al. prepared a series of bipyridine‐coordinated Ir motif‐linked porphyrins (M = Ni, Co, Fe, Mn, or Cu) loaded on carbon nanotubes, followed by high‐temperature pyrolysis to achieve heteronuclear DACs. The results showed that the M‐Ir dual‐site existed in an N_2_−M−N_2_−Ir−N_2_ configuration, the percentage of M‐Ir pairs and inter‐site distance were in the range of 66−70% and 2.43 to 2.55 Å, respectively.^[^ ^80^ ^]^ Wang et al. employed a metal‐ion recognition technique using inorganometallic cations and anions pairs ([M_A_L_x_]ⁿ⁺[M_B_L’_y_]ⁿ⁻) as precursors (Figure 4e). These pairs were sequentially adsorbed onto NC, relying on electrostatic interactions to ensure precise stoichiometry and close proximity between metal atoms. This method promoted the formation of N₂−M_A_−N₂−M_B_−N₂ motifs during pyrolysis, maximizing dual‐site generation. For Fe‐Sn pairs, 70% of the species form pairs with an interatomic distance of ≈2.85 Å (Figure 4f,g). These pairs exhibit highly effective ORR capability with a peak power density of 1.218 W cm⁻^2^ under 2.0 bar H₂‐O₂ conditions.^[^ ^81^ ^]^

#### 2.2.3 Leveraging π–π Stacking Interactions

**Figure 5:**
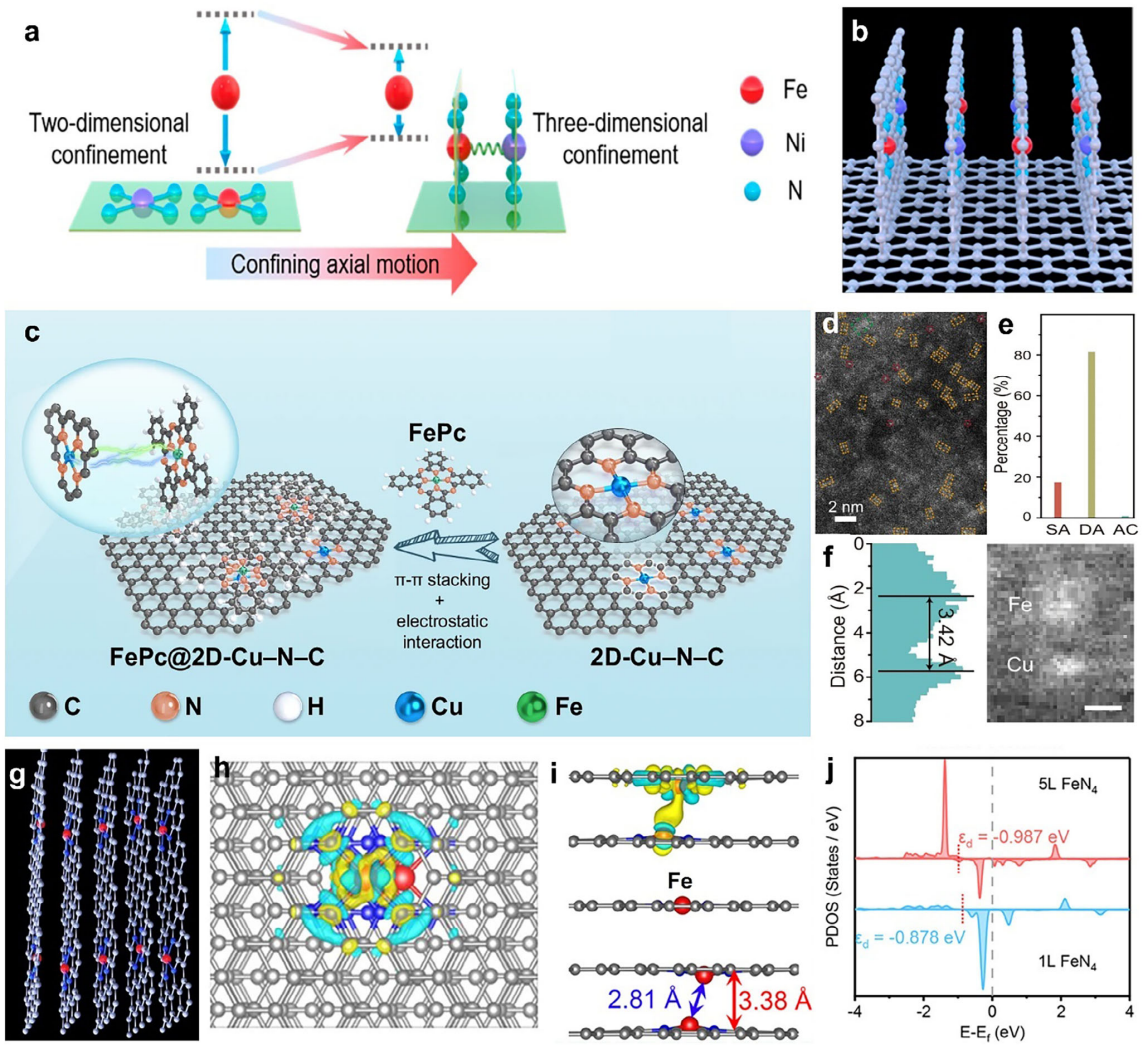
Precise regulation of the inter‐site distance between neighboring active sites through the fabrication of axial dual‐sites using 2D materials. a) Schematic diagram of the 2D and 3D confinement effects. b) Structural model of vertical graphene with limited Fe–Co dual‐site pairs. a,b) Reproduced with permission.^[^ ^82^ ^]^ Copyright 2023, American Chemical Society. c) The schematic synthesis of Fe–Co dual‐site pairs. d–f) Statistical study of metal‐metal pairs from AC‐HAADF‐STEM images. c‐f) Reproduced with permission.^[^ ^83^ ^]^ Copyright 2024, The Author(s), published by Nature Publishing Group. g) Structural model of several vertical planes. h, i) Charge density difference of five‐layered (5L) FeN_4_. j) PDOS of Fe in one‐layered (1L) FeN_4_ (light blue) and 5L FeN_4_ (orange). g‐j) Reproduced with permission.^[^ ^84^ ^]^ Copyright 2022, American Chemical Society.

In the case of planar dual‐sites, the coordination atoms and neighboring sites provide stabilization for metal atoms through a 2D confinement effect. However, this effect is restricted to the plane and does not prevent axial movement. To overcome this limitation, π‐π stacking interactions between 2D materials, such as graphene and phthalocyanine, can be employed to create dual sites with controlled axial distances. By employing vertically stacked graphene structures, single sites on adjacent layers can establish axial dual‐sites, thereby achieving a 3D confinement effect (**Figure** **5**a). This configuration enhances structural stability by restricting out‐of‐plane mobility.^[^ ^82^ ^]^ For instance, axial Ni‐Fe dual‐sites were synthesized by first creating 3D confined regions within vertically stacked graphene layers. These graphene layers, with interlayer spacings of 3.4 Å and abundant N defects, effectively anchored Ni and Fe atoms, resulting in a high concentration of axial dual‐sites (Figure 5b). Theoretical calculations revealed that the Ni sites vertically modulate the charge distribution of adjacent Fe sites, which lowers the *d*‐band center and weakens *CO intermediate adsorption. This inhibition of H₂ production during CO₂ electroreduction enables tunable syngas generation.^[^ ^82^ ^]^ In another instance, Fe phthalocyanine (FePc) molecules can be assembled onto positively charged 2D NCs loaded with single‐atom Cu through π–π stacking and electrostatic interactions (Figure 5c). This assembly forms Fe–Cu pairs with a separation of 3.4 Å (Figure 5d–f). The vertically stacked Fe–Cu configuration facilitates electron transfer, localizes charge polarization, and modulates substrate affinity. This architecture demonstrates outstanding potential for O₂‐dependent oxidative dehydrogenation in the metabolization of 1,4‐DHP.^[^ ^83^ ^]^ Additionally, film surface shrinkage has been utilized to induce the self‐assembly of M‐PC (M = Fe, Co, Cu) molecules via π‐π stacking interactions, forming one‐dimensional single‐atom arrays (Figure 5g). The structure comprises periodic stripes of vertical graphene layers, each embedded with metal atoms. Their DFT analysis revealed that following structural relaxation, the distance between the M sites decreases to 2.81 Å, suggesting strong interactions at the interplanar M sites within the stripes (Figure 5h,i). With the stacking of the layers, the *d*‐band center of the Fe atoms shifts from −0.878 to −0.987 eV, widening the energy gap between the *d*‐band center and the Fermi level (Figure 5j). This shift in the *d*‐band center reduces the adsorption energy for intermediates like *OOH, *O, and *OH, which consequently lowers the reaction overpotential.^[^ ^84^ ^]^

#### 2.2.4 Support Modification

Support materials with high surface areas, facile structural modification, and high porosity, such as zeolites, MOFs, and COFs, are particularly effective in promoting the uniform dispersion of dual‐sites. These materials feature tailored pore structures that allow metal atoms to remain well dispersion, preventing them from clustering and ensuring that the dual‐sites are maintained isolated and active. Additionally, the porosity and pore size of these supports can be finely adjusted to facilitate reactant accessibility to the active sites while maintaining site isolation.

Functional modification of these supports involves incorporating organic ligands/linkers that provide coordination sites for metal‐metal pairs, achievable through bottom‐up synthesis or post‐modification techniques. In heterogeneous catalysts involving porous materials, these ligands can stabilize metal active motifs and form part of the framework. They also control the distribution of active sites and can act as active sites themselves. A key challenge in preparing dual‐site catalysts is preventing metal aggregation. Auxiliary ligand design can effectively address this issue by spatially separating metal sites, either by forming stable metal‐based complexes at the nodes or on the surfaces of the channels. This separation mitigates aggregation and maintains atomic precision after thermal treatment, enhancing the functionality and efficiency of bimetallic catalysts.

The design and synthesis of catalysts with atomically precise active sites have transformed the field of catalysis, allowing for exceptional control over reactivity and selectivity. These catalysts, which range from SACs to multi‐atom systems like those involving metal‐metal pairs, exhibit highly tunable properties influenced by their coordination environments, inter‐site separation, and interactions with the support. Achieving atomic‐level precision necessitates innovative strategies to address challenges such as controlling dispersion, preventing aggregation, and optimizing the proximity of active sites. This precision enables the rational design of multifunctional catalysts, harnessing synergistic interactions between active motifs to drive complex reactions efficiently and sustainably across various catalytic processes.

##### Grafting Neighboring Active Sites on Zeolite Matrices

Microporous zeolites are well‐known for their ability to host isolated metal species through ion‐exchange with BASs, but isolated metal species on non‐acidic supports frequently do not exhibit comparable catalytic performance, highlighting the cooperative roles of nearby BASs.^[^ ^85^, ^86^ ^]^ A long‐standing challenge in this field has been the difficulty in establishing structure‐activity relationships due to the lack of high‐quality single crystals of modified zeolites. Fortunately, advancements in analytical techniques, such as the Rietveld refinement of synchrotron X‐ray powder diffraction (SXRD) and/or neutron powder diffraction (NPD) datasets, have enabled researchers to determine the presence of reaction substrate molecules adsorbed on the active sites. This helps the elucidation of the synergism, in terms of how probe molecules are activated in terms of bond distances and angles, between the neighboring active motifs.^[^ ^87^ ^]^

**Figure 6:**
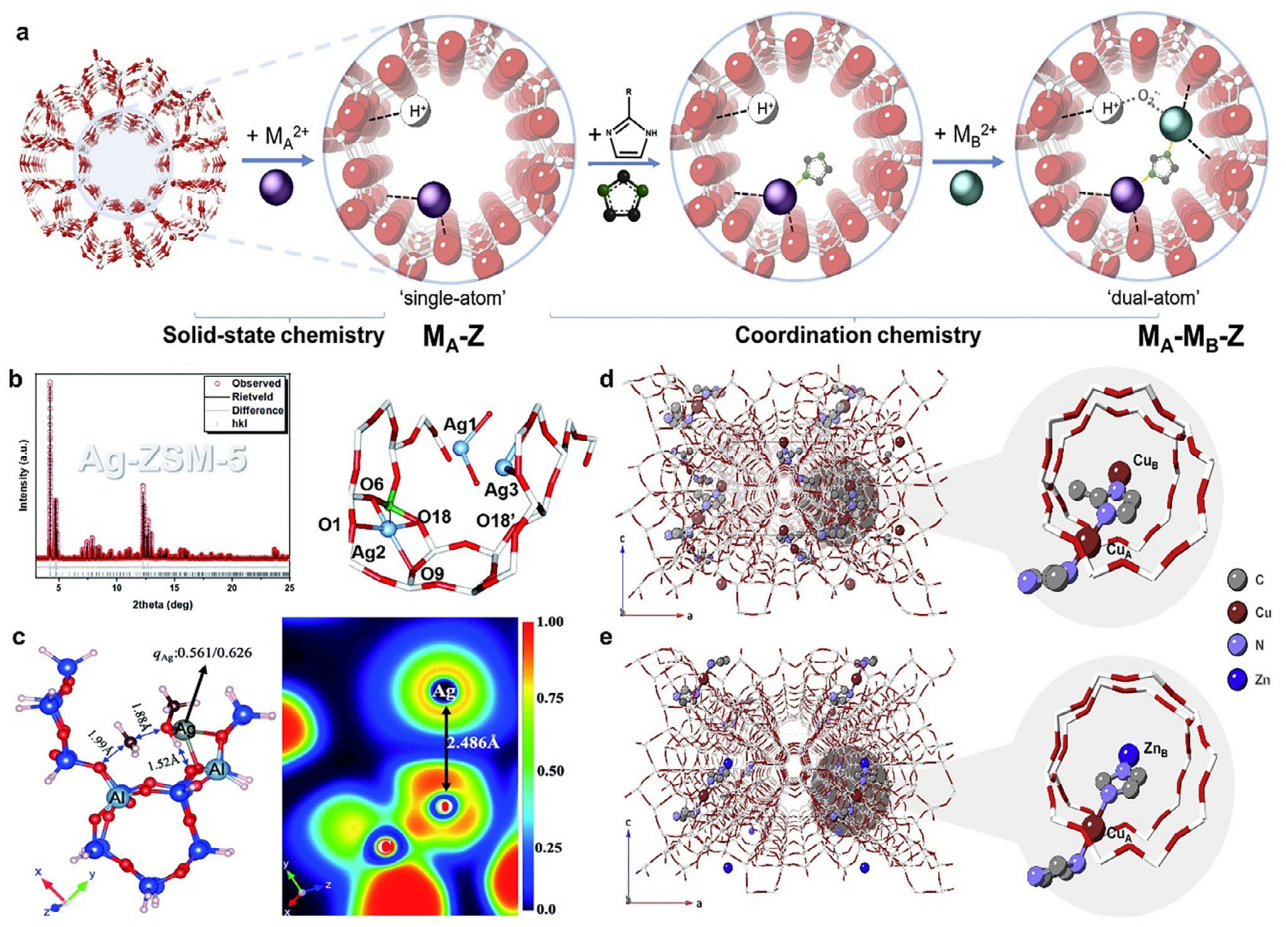
Grafting active site pairs on zeolite channels. a) Schematic illustration of a “step‐by‐step” assembly approach. Reproduced with permission.^[^ ^8^ ^]^ Copyright 2022, Elsevier Inc. b) SXRD and Rietveld refinement measured and corresponding refined crystal structure of Ag‐ZSM‐5. c) Transition state structure, charge value, and electron location function contour of methanol adsorption on Ag‐BAS pairs. b,c) Reproduced with permission.^[^ ^88^ ^]^ Copyright 2020, Royal Society of Chemistry. d) Atomic and structural elucidation of Cu‐Cu and e) Cu–Zn pairs in H‐ZSM‐5. Reproduced with permission.^[^ ^90^ ^]^ Copyright 2022, The Author(s), published by Nature Publishing Group.

Lin et al. explored the nature of the catalytically active sites in three metal‐modified H‐ZSM‐5 catalysts (M = Fe, Zn, and Ag). They proposed the formation of constrained metal‐BAS pairs on the basis of the interatomic distances between these active motif sites (**Figure** **6**b,c). The precise structure of these pairs enables a systematic examination of structure‐activity relationships in the activation of small molecules, such as methanol and water, providing insights into the initial chemical bond activation by metal‐BAS pairs within the zeolite framework.^[^ ^88^ ^]^ Wun et al. also synthesized a series of zeolites with varying metal‐BAS pairs (M = Fe, Cu; X = B, Al, Ga) with delicate control over their quantities and respective distribution. Combined SXRD and theoretical evidence revealed the synergism and the respective contribution of the active pairs of metal‐BAS within molecular distances. By employing two model catalytic reactions that simultaneously utilize the two active motifs in proximity, the synergistic cooperativity between the metal‐BAS pairs has been systematically studied.^[^ ^89^ ^]^ This work offers a clear molecular model that directly pinpoints the interaction between the substrate molecules and the support matrix, which notably enhances the understanding of the synergism between the active motifs.

Additionally, organic linkers can effectively connect two active sites, allowing for precise control over the geometric distance between them. Multidentate and dibasic linkers are ideal choices, as they can bind multiple metal motifs simultaneously. These ligands typically feature multiple coordination sites, enhancing stability and flexibility in the arrangement of active sites. By utilizing multidentate linkers, researchers can tune the spatial arrangement of metal centers, crucial for optimizing catalytic activity and selectivity. This strategic design also facilitates synergistic interactions between active sites, boosting overall catalytic efficiency.

Inspired by the native Cu,Zn‐containing superoxide dismutase, Wun et al. have developed a strategy to construct atomically precise bimetallic ligand‐mediated metal ensembles within zeolites (Figure 6a). This strategy emphasizes the critical importance of atomic‐level structural accuracy in catalytic systems. Utilizing the underlying principles of coordination chemistry and solid‐state chemistry, M^2^⁺ ions can be anchored to BASs in zeolites via conventional ion‐exchange. Rigid and dibasic imidazolate linkers are then employed to sequentially assemble 3*d* transition metal ions (e.g., Co^2^⁺, Ni^2^⁺, Cu^2^⁺, and Zn^2^⁺), which can ensure a 1:1:1 metal:linker:metal coordination without excessive chelation/metalation (Figure 6d,e). Owing to the high crystallinity of the zeolite support, the precise crystallographic locations of these metal sites with respect to the host have been determined by resonant X‐ray diffraction. The catalysts feature two ligand‐mediated extra‐framework metal sites within the zeolitic micropores with a hierarchical micro‐mesoporous structure, where the two metal sites are separated by ≈5.7 Å. This precise arrangement of metal‐metal pairs enables the efficient dehydrogenative cross‐coupling reactions of unprotected phenols and amines and the Huisgen azide‐alkyne cycloaddition “click” reaction under mild conditions. The promotional effect has been attributed to a “co‐adsorption‐co‐activation” molecular model for the reaction substrates separately offered by the two metal sites within the ligand‐mediated metal ensembles.^[^ ^6^, ^8^, ^90^, ^91^ ^]^

##### Anchoring Neighboring Active Sites in MOF Pores

MOFs serve as versatile platforms for designing active sites with complex catalytic active motifs by leveraging their porous structures and tunable metal species. Atomically precise active sites can be constructed through bottom‐up synthesis at the nodes or by post‐modification linkers for grafting sites on the pore surfaces.

**Figure 7:**
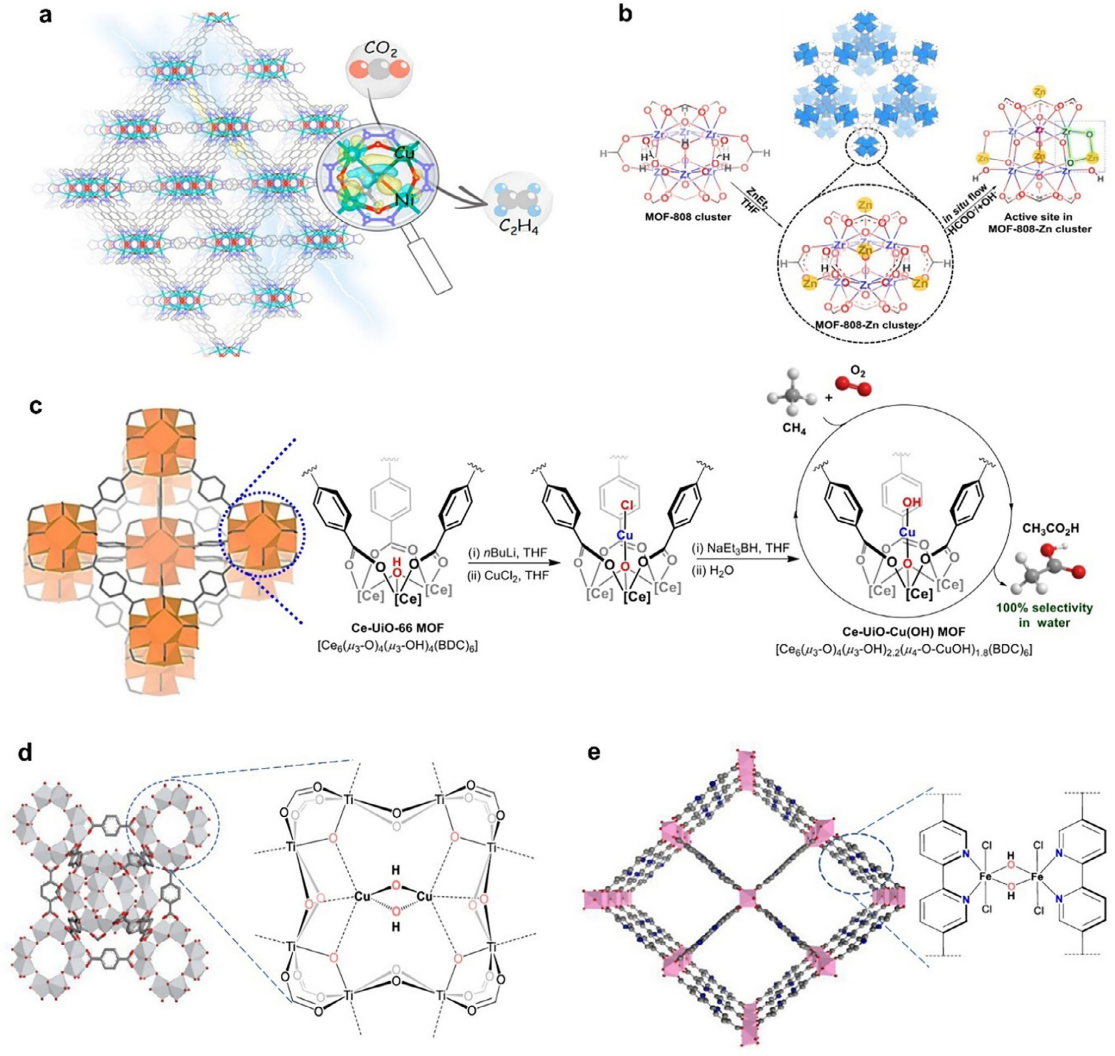
Anchoring metal‐metal pair active sites on MOF pores. a) Structural model of the cage structure in the Cu_1_Ni‐BDP MOF and the calculated charge density difference of the Cu_1_Ni cubic cluster. Reproduced with permission.^[^ ^94^ ^]^ Copyright 2023, American Chemical Society. b) Scheme showing the synthesis of MOF‐808‐Zn and the active site of Zr^4+^─O─Zn^2+^. Reproduced with permission.^[^ ^95^ ^]^ Copyright 2021, American Chemical Society. c) Coordinatively unsaturated mono‐Cu(II) hydroxyl catalyst supported by Ce‐MOF nodes for direct conversion of methane to acetic acid in water using molecular oxygen as the oxidant. Reproduced with permission.^[^ ^98^ ^]^ Copyright 2023, American Chemical Society. d) Post‐synthetic modification of MOF‐253 to construct [(bpy)Fe(*µ*_2_‐OH)]_2_ catalytic centers. e) MOF‐based artificial monooxygenase Ti_8_‐Cu_2_. d‐e) Reproduced with permission.^[^ ^101^ ^]^ Copyright 2023, American Chemical Society.

The abundance of N sites in aromatic N‐heterocycles facilitates the formation of robust metal─N bonds, making them ideal for constructing atomically precise multi‐nuclear metal‐based MOFs. For example, 1H,1,2,4‐triazole (Htrz) has been applied as a linker for the synthesis of [Cu_3_(*µ*_3_‐OH)(*µ*_3_‐trz)_3_(OH)_2_(H_2_O)_4_]·xH_2_O, formed by interlinking cyclic trinuclear [Cu_3_(*µ*_3_‐OH)(*µ*_3_‐trz)_3_]^2+^ clusters. Each Cu(II) atom is octahedrally coordinated with three N atoms from the trz^−^ ligands, with an optimal intracluster Cu···Cu distance of 3.3 Å facilitating *CO coupling to generate the *COCOH intermediate, crucial for the subsequent steps for the production of C_2+_ products.^[^ ^92^ ^]^ Another notable example is the use of benzotriazolate, which exhibits a pyrazolate coordination mode allowing it to bind with a pair of Cu(I) ions, forming a Cu(I) site with a Cu···Cu distance of 3.52 Å. This configuration serves as a neighboring active site that facilitates C−C coupling by lowering the Gibbs free energy barrier for the CO_2_RR toward C_2+_ products. Additionally, the uncoordinated N atoms of benzotriazolate in close proximity act as proton relays, which enhances the overall catalytic activity through synergistic effects.^[^ ^93^ ^]^ Recent advancements have also led to the successful design and synthesis of asymmetric Ni/Cu catalytic sites within pristine CuNi‐1,4‐benzenedipyrazolate MOF (**Figure** **7**a). The proximity of neighboring N sites results in a measured distance of 2.8 Å between adjacent Cu and Ni sites, allowing them to function synergistically. This synergy lowers the energy barrier for forming *COH−COH, which is the rate‐limiting step in the conversion of CO_2_ to C_2_H_4_. Charge density difference analysis revealed notable charge accumulation and depletion within the hybrid Cu‐Ni clusters, highlighting their asymmetric electronic structure and orbital interactions.^[^ ^94^ ^]^

Moreover, the ligands on the surface of MOF pores can be fully utilized through post‐modification techniques such as ligand exchange, allowing for the functionalization and modification of their pores to prepare synergistic catalysts with metal‐metal pair active sites. For instance, Zhang et al. constructed well‐defined Zr⁴⁺−O−Zn^2^⁺ sites adjacent to each other in MOF‐808 through the deprotonation of *µ*₃‐OH using ZnEt₂ (Figure 7b). The authors revealed that the Zn^2^⁺ and the surrounding O site have formed a Lewis acid‐base pair for heterolytic H₂ cleavage. This interaction generates Zn^2^⁺−H^δ⁻^ and Zr⁴⁺−O−H^δ⁺^, which are critical for further CO₂ adsorption and conversion.^[^ ^95^ ^]^ Contributions from Manna's group have advanced this field through the design and synthesis of heterogeneous single‐site late 3*d* metal hydroxide catalysts. These catalysts form active M_A_‐M_B_(OH) motifs, such as Ce‐Co^2^⁺(OH), Ni^2^⁺(OH), Cu^2^⁺(OH), and Al‐Fe^3^⁺(OH)₂, supported by Ce or Al MOF nodes (Figure 7c). For instance, Ce‐Co^2^⁺(OH) was synthesized via stepwise reactions. The *µ*₃‐OH groups of the nodes were deprotonated with *n*‐BuLi, followed by salt metathesis with CoCl₂ in tetrahydrofuran at room temperature to yield Ce‐CoCl. Further reduction with NaEt₃BH yielding Ce‐CoH, which was subsequently converted to Ce‐Co(OH) upon exposure to water.^[^ ^96^ ^]^ These MOF‐supported catalysts demonstrated remarkable efficiency in selectively oxidizing CH₄ to CH₃OH, CH₃CO₂H, and CH₃CH₂OH using O₂ or H₂O₂. The precise binding sites, oxidation states, and reactive intermediates of M(OH) within the MOF framework were resolved using synchrotron radiation techniques, enabling atomic‐level structural insights. This approach provides a new avenue for mechanistic studies of catalysis at the molecular level. Encapsulating single metal hydroxyl sites within porous MOFs offers a promising strategy to develop efficient, earth‐abundant catalysts for methane functionalization into valuable chemicals.^[^ ^96^, ^97^, ^98^, ^99^ ^]^ Feng et al. developed an artificial Cu₂ monooxygenase based on a Ti‐based MOF. Deprotonation of hydroxide groups on secondary building units enabled metalation with Cu^+^ pairs, forming a binuclear Cu^2+^−(*µ*₂‐OH)₂ −Cu^2+^ cofactor upon oxidation by O₂ (Figure 7d). The closely spaced Cu₂ sites stabilized the Cu─O₂ adduct, reducing the free energy for O─O bond cleavage by 6.6 kcal mol^−1^ compared to Cu_1_ sites, resulting in greatly enhanced catalytic activity. Cu^2+^−(*µ*₂‐OH)₂−Cu^2+^ demonstrated exceptional performance in diverse monooxygenation reactions, achieving turnover numbers up to 3450 with the presence of co‐reductants.^[^ ^100^ ^]^ Wang et al. introduced a self‐adaptive, bipyridine‐based MOF‐253 for constructing a [Fe^3^⁺(*µ*₂‐OH)]₂ artificial monooxygenase (Figure 7e). The parallel alignment and optimal spacing of bipyridine linkers within MOF‐253 facilitate the formation of binuclear [(bpy)Fe^3^⁺(*µ*₂‐OH)]₂ catalytic centers that mimic natural monooxygenases. These Fe₂‐based neighboring pairs demonstrate high catalytic performance in C−H oxidation and epoxidation reactions using O₂ as the oxidant.^[^ ^101^ ^]^ Similarly, Li et al. incorporated a flexible metal‐binding pyridylmethylamine moiety into the UiO‐67 framework, subsequently installing Cu₂ sites to create Cu₂O₂ neighboring motifs. This configuration was confirmed through spectroscopic studies and DFT calculations. The enhanced catalytic performance observed in the aerobic Chan‐Lam reaction, characterized by high activity and recyclability, can be attributed to the remote synergistic effect of the paired Cu site, which binds molecular dioxygen and collaboratively cleaves the O═O bond.^[^ ^102^ ^]^ Furthermore, Li et al. developed a bioinspired photocatalyst featuring a flexible EDTA‐chelated Cu^2^⁺‐Ni^2^⁺ pair incorporated into MOF‐808. The spatial configurations of this catalyst evolve continuously with diverse C_1_ intermediates in a self‐adaptive manner during multistep photocatalytic CO₂ reduction, achieving CO₂‐to‐CH₄ photoreduction with CH₄ selectivity of 99.4% (electron basis) and 97.5% (product basis).^[^ ^103^ ^]^

Overall, these examples illustrate some innovative strategies employed in the design and synthesis of metal‐metal pair sites within MOFs, highlighting their potential for advancing catalysis and enabling efficient transformations of small molecules.

##### Integrating Metal–Metal Pair Active Sites in COFs

In addition to MOFs, COFs possess inherent porosity that allows them to immobilize metal ions as active sites, resulting in the formation of metallo‐covalent organic frameworks (M‐COFs). Tetradentate ligands such as N, N’‐Bis(salicylidene)ethylenediamine (H_2_salen) are significant in coordination chemistry due to their ability to stabilize metal ions across various oxidation states, leading to the development of metallosalen COFs as promising candidates for advanced heterogeneous catalysis.^[^ ^104^, ^105^ ^]^

**Figure 8:**
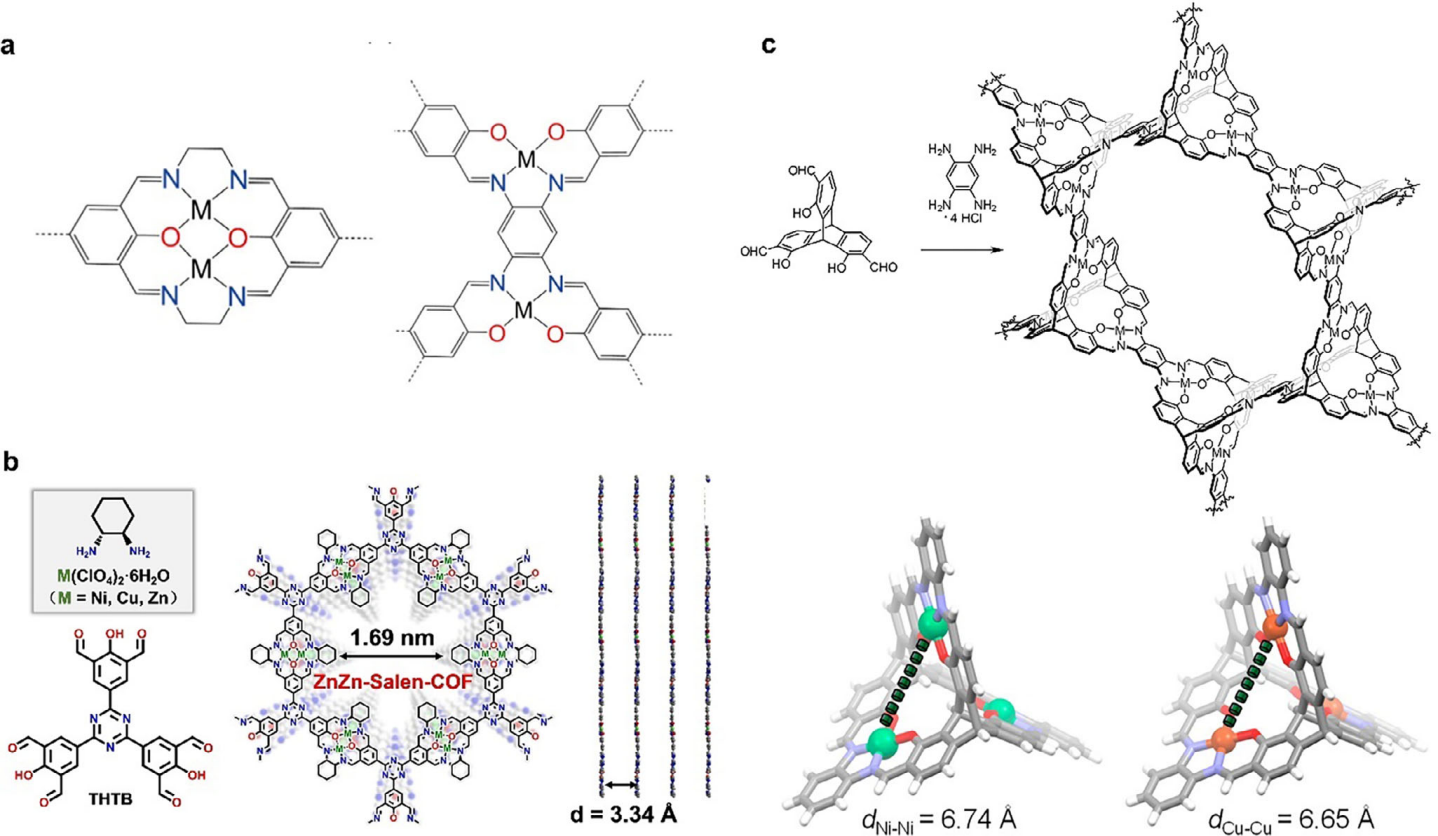
The integration of metal‐metal pair active sites in COFs with salen ligands. a) Two configurations of M_1_M_2_‐Salen‐COF. Reproduced with permission.^[^ ^104^ ^]^ Copyright 2024, The Authors. Interdisciplinary Materials published by Wuhan University of Technology and John Wiley & Sons Australia, Ltd. b) Synthetic Scheme for the preparation and the structures of ZnZn‐Salen‐COF. Reproduced with permission.^[^ ^110^ ^]^ Copyright 2024, Wiley‐VCH GmbH. c) Syntheses of model compounds and the network materials Ni_3_‐MaSOF and Cu_3_‐MaSOF from trissalicylaldehyde. Single‐crystal X‐ray structures of trinuclear metal complexes. Reproduced with permission.^[^ ^109^ ^]^ Copyright 2019, American Chemical Society.

Conventional salen ligands possess only four coordination sites (2 × N and 2 × O), restricting their capacity to stabilize isolated transition metal ions and preventing the formation of metal‐metal pairs that could boost synergistic catalytic performance. In recent years, advancements in the modification of salen ligands have led to derivatives with increased coordination sites, allowing for the accommodation of two neighboring metal ions (**Figure** **8**a). Zang et al. developed a 2D salen COF featuring double vacancies, incorporating Cu into the framework with an N₂−Cu−O₂−Cu−N₂ configuration for photocatalytic hydrogen evolution. The resulting Cu_2_‐salen‐HDCOF features a conjugated porous structure with densely packed Cu₂ active motifs, enhancing photogenerated charge transfer to surface redox centers while minimizing exciton recombination.^[^ ^106^ ^]^ Similarly, Zhou et al. synthesized gram‐scale Zn₂‐based metallosalen‐COFs through a one‐step, vacuum‐free autoclave process for efficient CO₂ photoreduction. The coordination environment of N₂−Zn−O₂−Zn−N₂, confirmed by XAFS spectroscopy, was pivotal to their functionality.^[^ ^107^ ^]^ Zhang et al. reported a Robson‐type Zn_2_ catalyst for poly(ethylene terephthalate) (PET) degradation. The resulting Zn_2_‐based COF features a *µ*_2_‐O bridged Zn‐Zn structure with a distance of 3.2 Å, which is comparable to that observed in binuclear metallohydrolases (3.5 ± 0.5 Å), as confirmed by single‐crystal X‐ray diffraction (SCXRD) (Figure 8b). The increased Lewis acidity of Zn(II) in the *µ*_2_‐O bridged Zn_2_ sites enhances its ability to bind the carbonyl group in PET, activating it for nucleophilic attack and resulting in the formation of a six‐membered ring (Zn─O─C─O─Zn─*µ*_2_‐O). Key steps in the hydrolysis of PET facilitated by the binuclear catalyst include the co‐adsorption of substrates at the adjacent zinc sites and the stabilization of a six‐membered intermediate.^[^ ^108^ ^]^ Elbert et al. developed two *C*_3_‐symmetrical M‐COFs featuring Ni_3_ and Cu_3_ salphen complexes, achieving metal‐to‐metal distances of d_Ni‐Ni_ = 6.74 Å and d_Cu‐Cu_ = 6.65 Å, as confirmed by SCXRD (Figure 8c). These distances facilitate synergistic coordination with CO_2_, suggesting that the incorporation of trinuclear salphen metal complexes into porous structures can create free ligation sites on the copper centers through appropriate activation methods.^[^ ^109^ ^]^

Metal(M)‐COFs provide integrated active metal sites with well‐defined coordination environments, enabling precise structure‐activity relationship studies. These features position M(salen)‐COFs as versatile platforms for designing next‐generation catalytic materials.

## 3 Applications of Neighboring Active Motifs

In heterogeneous catalysis, the precise spatial arrangement and electronic interactions of neighboring active motifs can greatly enhance catalytic activity and selectivity. These motifs, including metal‐acid (Brønsted/Lewis) pairs, metal‐base pairs, and metal‐metal pairs, facilitate cooperative effects that activate substrates, stabilize intermediates, and lower reaction barriers (**Table** **1**). Such synergistic interactions result in improved efficiency, higher selectivity, and better control over reaction pathways. This section presents key examples showcasing how rational design and integration of neighboring active motifs drive advancements across various catalytic processes. By elucidating the mechanisms of these motifs, researchers can tailor catalysts for reactions like hydrocarbon transformations, CO₂ conversion, biomass upgrading, and so forth. These findings provide a framework for developing next‐generation multifunctional catalysts with unmatched efficiency.

**Table 1:** Synergistic effects of dual‐site catalysts on performance enhancement.

Catalysts	Active motifs	Reactions	Mechanisms	Performances^a)^	Techniques
Mo₁Al/MgO^[^ ^111^ ^]^	Metal site: Mo LAS: Al	Depolymerization	Al activates lignin and stabilizes intermediates, Mo cleaves *β*‐aryl ether bonds	Monophenolic yield Mo₁Al/MgO:46% Mo_1_/MgO:26%	HAADF‐STE, XANES, DFT, et al.
Ru/Nb₂O₅^[^ ^112^ ^]^	Metal site: Ru LAS: NbO_x_	Hydrogenolysis of aromatic plastic waste	Ru prevents arene hydrogenation, NbO_x_ activates C−O bonds, BASs cleave C−C bonds	Arenes yield Ru/Nb₂O₅: 75–85 %; Ru/ZSM‐5: ≈10%	DRIFT, XANES, DFT, et al.
Pt_1_/Co₂AlO₄^[^ ^113^ ^]^	Metal site: Pt BAS: Co─O(H)−Al	Hydrodeoxygenation of 5‐HMF to DMF	Pt activates C═O and cleaves C–OH, BAS dissociates C─O bonds	5‐HMF to DMF yield Pt_1_/Co₂AlO₄: >99%; Co_2_AlO_4_: 11%;	In situ XAFS, in situ FTIR, AC‐ STEM, et al.
Cu/M@SiO_2_ (M = Ti, Zr, Hf, Nb, or Ta)^[^ ^114^ ^]^	Metal site: Cu LAS: M (Ti)	CO_2_ to CH_3_OH	LAS stabilizes intermediates,Cu activates CO_2_,M LASs enhance CH_3_OH formation	CH_3_OH rate [µmol (g_Cu_ s)^−1^] Cu/Ti@SiO_2_: 18.1; Cu/SiO_2_: 3.61	IR, TEM, Solid state CP‐MAS NMR, et al.
Pt/CeO₂@ZSM‐5^[^ ^115^ ^]^	Metal site: Pt BAS: ZSM‐5	Furfural into cyclopentanone	CeO_2_ enhances metal/acid interactions, Pt hydrogenates FFL‐to‐FAL, BAS converts FFL‐to‐CPO	Cyclopentanone selectivity Pt/CeO₂@ZSM‐5: 97.2%; Pt/ZSM‐5: 61.4%	EXAFS, in situ ATR‐IR, DFT et al.
Pt@S‐1+ *β* zeolite^[^ ^116^ ^]^	Metal site: Pt BAS: *β* zeolite	Conversion of PE into naphtha	Acid cracks/isomerizes PE, Pt hydrogenates olefins	Naphtha yield Pt@S‐1+*β* zeolite: 89.5%; Pt@S‐1: ≈70%	HAADF‐STEM, EXAFS, DFT, et al.
Pd/NUS‐SO_3_H^[^ ^117^ ^]^	Metal site: Pt BAS: NUS‐SO_3_H	Hydrodeoxygenation of 5‐HMF	Pt hydrogenates, BAS dehydrates/etherifies, LAS activates glucose	5‐HMF conversion Pd/NUS: 67.5 %; Pd/NUS‐SO_3_H: 92.1 %	HAADF‐STEM, FTIR, XPS, PXRD et al.
MOF‐OTf‐Pd^NP[^ ^118^ ^]^	Metal site: Pd LAS: Al_2_(µ_2_‐OTf)	Dehydroalkoxylation–hydrogenation	Al₂(µ₂‐OTf) cleaves C─O bonds, Pd hydrogenates C═C bonds	Menthane yield MOF‐OTf‐Pd^NP^:80%; MOF‐OH‐PdCl_2_:22%	PXRD, TEM, TGA, ICP‐MS, IR, XAS et al.
ADD‐Fe‐Al^[^ ^119^ ^]^	Metal site: Fe LAS: Al	Oxidative carboxylation of olefins	Al boosts olefin oxidation, Fe shifts the equilibrium to epoxidation	Conversion ADD‐Fe‐Al: ∼97% AD‐Fe: ∼62%	XANES, EXAFS, HADDF‐STEM, et al.
M/X‐Z (M = Fe, Cu) (X = Al, Ga, B)^[^ ^89^ ^]^	Metal site: Cu, Fe BAS: Al, Ga, B	Oxidation of 4‐chlorostyrene	Synergistic cooperation between different metal sites and BASs	yield of 4‐chlorobenzaldehyde Fe/Al‐Z: 1.6 mmol Fe/B‐Z: 0.4 mmol	EXAFS, SXRD, TPD, DFT, et al.
*ms*‐NiAl‐P350^[^ ^120^ ^]^	FLP: Ni(─O─Al)···P	Convert soybean oil to diesel‐range hydrocarbons	Ni(─O─Al)···P is a critical factor in achieving coke‐ and sintering‐resistant activity during hydrotreating reactions.	methyl laurate conversion *ms*‐NiAl‐P350: 85–88%: *ms*‐NiAl: significant drop from 80%.	HAADF‐STEM, DFT, EXAFS, et al.
InOOH‐100^[^ ^121^ ^]^	FLP: In···In‐OH	Urea synthesis from N_2_ and CO_2_	InOOH nanocrystals’ FLPs enable targeted adsorption and activation through orbital interactions.	Urea yield rate (mmol h^−1^ g^−1^) InOOH‐100: 6.85; InOOH‐100 poisoned by SCN^−:^ ≈2	HRTEM, Raman spectroscopy, FTIR, NMR, et al.
ZnIn_2_S_4_/In(OH)_3–_ *_x_* ^[^ ^122^ ^]^	FLP: In‐□···In‐OH	CO_2_RR	ZnIn_2_S_4_ harvests the light and In(OH)_3–_ *_x_* with FLPs activate the CO_2_.	CO formation rate (µmol g^−1^ h^−1^) ZnIn_2_S_4_/In(OH)_3–_ *_x_*: 1945.5; ZnIn_2_S_4_: ≈700	HAADF‐STEM, TPD, Py‐IR, EPR, in situ FTIR, DFT, et al.
c‐TiO_2_@a‐TiO_2‐x_(OH)_y_^[^ ^123^ ^]^	FLP: HO‐Ti‐[O]‐Ti	CO_2_RR	SFLPs enhance solar photon harvesting, photothermal effects, and electron‐hole dynamics for efficient H_2_/CO_2_ reactions.	CO formation rate (mmol g^−1^ h^−1^) c‐TiO_2_@a‐TiO_2‐x_(OH)_y_: 5.3; c‐TiO_2_: ≈0.015	DRIFTS, ^1^H‐MAS‐NMR, HAADF‐STEM, et al.
W_SA_‐PCN^[^ ^124^ ^]^	FLP: N···W_SA_	CO_2_RR	W (LA) enables π back‐donation to CO_2_, N (LB) facilitates electron transfer, creating a “push–push” effect.	CO formation rate (µmol g^−1^ h^−1^) W_SA_‐PCN: 46.7; WNP‐PCN: ≈25	XANES, EXAFS, AC‐HAADF‐STEM, TPD Py‐IR, DFT, et al.
Pt/TiN_x_O_y_^[^ ^125^ ^]^	FLP: Ti−NH_2_···Ti(L)	CO_2_RR	Ti‐NH_2_ (LB) and low‐valent Ti (LA) synergistically activate molecules, enhancing light‐assisted reverse water‐gas shift reaction	CO formation rate (mmol g^−1^ h^−1^) Pt/TiN_x_O_y_: >10 Pt/TiO_2_: ≈3	HRTEM, HAADF‐STEM, PXRD, DRIFTS, DFT, et al.
Fe_2_/NC^[^ ^126^ ^]^	N₂─Fe─N₂─Fe─N₂ (0.30 nm)	ORR	Extended Fe···Fe spacing enhances *OOH formation and ORR activity	ORR energy barrier Fe_2_/NC: 0.71 eV; Fe_2_/NC‐S (0.25 nm Fe···Fe): 0.87 eV	XAFS, HRTEM, DFT, XAFS O_2_‐TPD, DRIFTS et al.
Fe_2_/N_6_‐S^[^ ^127^ ^]^	N₂─Fe─N₂─Fe─N₂ (≈ 0.25 nm)	ORR	Fe₂ activates O₂, lowers d‐band, releases OH*; S‐doping accelerates OOH* formation	ORR activity *E*_1/2_ Fe_2_/N_6_‐S: 0.921 V; FeN_4_: 0.888 V	DFT, HR‐TEM, HAADF‐STEM, XAS, et al.
Fe_2_/NPC^[^ ^9^ ^]^	N₃─Fe─Fe─N₃ (<0.26 nm)	CO₂RR	e‐Fe pairs activate CO₂ and lower CO desorption barriers	FE_CO_ at −0.6 V Fe_2_/NPC: 96.0%; Fe_1_NPC: 82.0%	HAADF‐STEM, XAS, TPD, et al.
Cu_2_/C_2_N^[^ ^128^ ^]^	N₂─Cu₂─N₂ (0.24 nm)	CO₂RR	Adjacent Cu sites dynamically reposition on C₂N to boost CO adsorption and dimerization	FE_C2+_ Cu_2_/C_2_N: 90.8%, Cu_1_‐C_2_N: 0	in situ ATR‐SEIRAS and XAS, et al.
Fe_2_/NG^[^ ^129^ ^]^	N₃─Fe─Fe─N₃ (0.23 nm)	N₂ORR	Modulated structure bends N₂O for 8e^−^ NH₃ synthesis	FE_NH₃_ at −0.6 V Fe₂/NG:77.8%; FeN_4_: ≈45%	AC‐HAADF‐STEM, XANES, EXAFS, et al.
Pt_2_/mpg‐C_3_N_4_^[^ ^130^ ^]^	O_x_─Pt─Pt─N_2_ (0.255 nm)	Hydrogenation reaction	Pt₂’s diatomic nature enhances H₂ dissociation and aniline desorption.	nitrobenzene conversion Pt_2_/mpg‐C_3_N_4_: >99%; Pt_1_/mpg‐C_3_N_4_: 23%	AC STEM, XAFS, DFT, et al.
Pt_1_Ru_1_/NCNTs^[^ ^131^ ^]^	N_x_‐Pt‐Ru‐C_y_ (0.24 nm)	HER	The interaction between H and Ru could be modulated by the Pt through the synergetic effect.	Mass activity for HER at *η* = 0.05 V (A mg^−1^) Pt_1_Ru_1_/NCNTs: 23.1: Pt/C: 0.43	HAADF‐STEM, XANES, EXAFS, et al.
Zn_1_Ru_1_/PCN^[^ ^132^ ^]^	N₃─Zn─Ru─N₃ (0.25 nm)	HER	The Ru site reduces H^+^ to H* and the Zn site acts as the H* desorption site.	H_2_ yield (mmol g_Ru_^−1^) Zn_1_Ru_1_/PCN: 4138 Ru‐PCN: ≈1000	HAADF–STEM, XAS, DFT, et al.
Pt_1_Ni_1_/C_3_N_4_^[^ ^133^ ^]^	N₂─Ni─O₂─Pt─O₂ (0.22 nm)	Dehydrogenation of AB	Metal synergy boosts H_2_ evolution via water dissociation and B─H cleavage	Yield (mol_H2_ mol_Pt_^−1^ min^−1^) Pt_1_Ni_1_/C_3_N_4_: 1444: Pt_1_/C_3_N_4_: ≈110	HAADF‐STEM, EXAFS, et al.
Pd_1_Mn_1_/NC^[^ ^134^ ^]^	N₂─Pd─N₂─Mn─N₂ motifs (0.23 nm)	Hydrogenation reaction	Mn‐to‐Pd electron transfer enhances adsorption and lowers hydrogenation barriers.	TOF (mol_C═C_mol_Pd_^−1^ min^−1^) Pd_1_Mn_1_/NC: 218 Pd_1_/NC: 24	HAADF‐STEM, PXRD, XAS, DFT, TPD, et al.
Fe_1_Mn_1_/NC^[^ ^135^ ^]^	N₂─Fe─N₂─Mn─N₂ (0.23 nm)	ORR	The catalytic activity arises from the cooperative effect of the Fe‐Mn pairs aiding *OOH dissociation.	ORR *E*_1/2_ (V) Fe_1_Mn_1_: 0.922 Mn_1_: 0.810	AC HAADF‐STEM, XANES, EXAFS, et al.
Co_1_Ni_1_/NC^[^ ^136^ ^]^	N_3_─Co─Ni─N_3_ (0.22 nm)	ORR	Co‐Ni synergy optimizes adsorption/desorption and lowers reaction barriers.	ORR: *E*_onset_ & *E*_1/2_ (V) Co_1_Ni_1_: 0.88 & 0.76: CoNi‐NPs:0.78& 0.64	AC‐STEM, XAS, DFT, SEM, XPS, et al.
Ni_1_Fe_1_‐DASC^[^ ^137^ ^]^	N₂─Fe─N₂─Ni─N₂ (0.24 nm)	CO₂RR and OER	Ni‐Fe pairs modulate electronic states to weaken intermediate binding	FE_CO_ Ni_1_Fe_1_: 94.5%; Fe_1_: ≈25%	XRD, SEM, AFM, HAADF‐STEM, et al.
Fe_1_Cu_1_/HNG^[^ ^138^ ^]^	N₂─Fe─Cu─N₂ (0.23 nm)	NO_3_^−^RR	Fe‐Cu dual‐sites boost NO₃⁻ conversion by weakening N−O bonds.	FE_NH3_ at −0.3 V Fe_1_Cu_1_/HNG: 92.51% Fe_1_/HNG: 70%	HAADF‐STEM, XANES, EXAFS, et al.
Cu‐M‐Y (M = Co, Ni)^[^ ^6^ ^]^	Cu‐M	C(sp^2^)─N Coupling Reaction	Cu‐Co synergy enhances substrate adsorption vs other metal pairs.	Isolated yield (%) Cu─Co─Y: 81; Cu_1_─Y: trace	EXAFS, SXRD, EPR, TEM, DFT, et al.

^a)^ *E*_1/2_: half‐wave potential; *E*_onset_: onset potential; *η*: overpotential. All potentials were referenced to the reversible hydrogen electrode (RHE).

The table uses color‐coded shading to classify different catalytic pairs: green represents metal‐acid pairs (Brønsted/Lewis), blue represents metal‐base pairs, yellow represents homonuclear metal‐metal pairs, and gray represents heteronuclear metal‐metal pairs.

### 3.1 Metal‐Acid (Brønsted/Lewis) Pairs

Metal‐acid pairs are vital in heterogeneous catalysis, offering synergistic interactions that enhance substrate activation, bond formation, and intermediate stabilization. BASs function by protonating substrates, reducing activation energy, and facilitating tandem reactions while regenerating active motifs. The strength of these sites can be tuned via the conjugate base, following Brønsted‐Lowry principles. Conversely, LASs activate substrates by electron withdrawal without proton transfer, involving elements such as B, Al, Sn, or TMs. LASs can polarize substrates, form additional adsorption sites, and modulate reactivity. The strength of the LASs can be modulated through ligands or anions to adjust the lowest unoccupied molecular orbital energy level. The spatial arrangement and acidity of metal‐acid pairs are critical for catalytic performance. Proximity between the paired sites ensures rapid interactions, while excessive separation reduces efficiency. Structural supports, tailored ligands, and confined spaces in materials like metal oxides, zeolites, MOFs, and C‐based materials optimize spacing and tune acidity.

**Figure 9:**
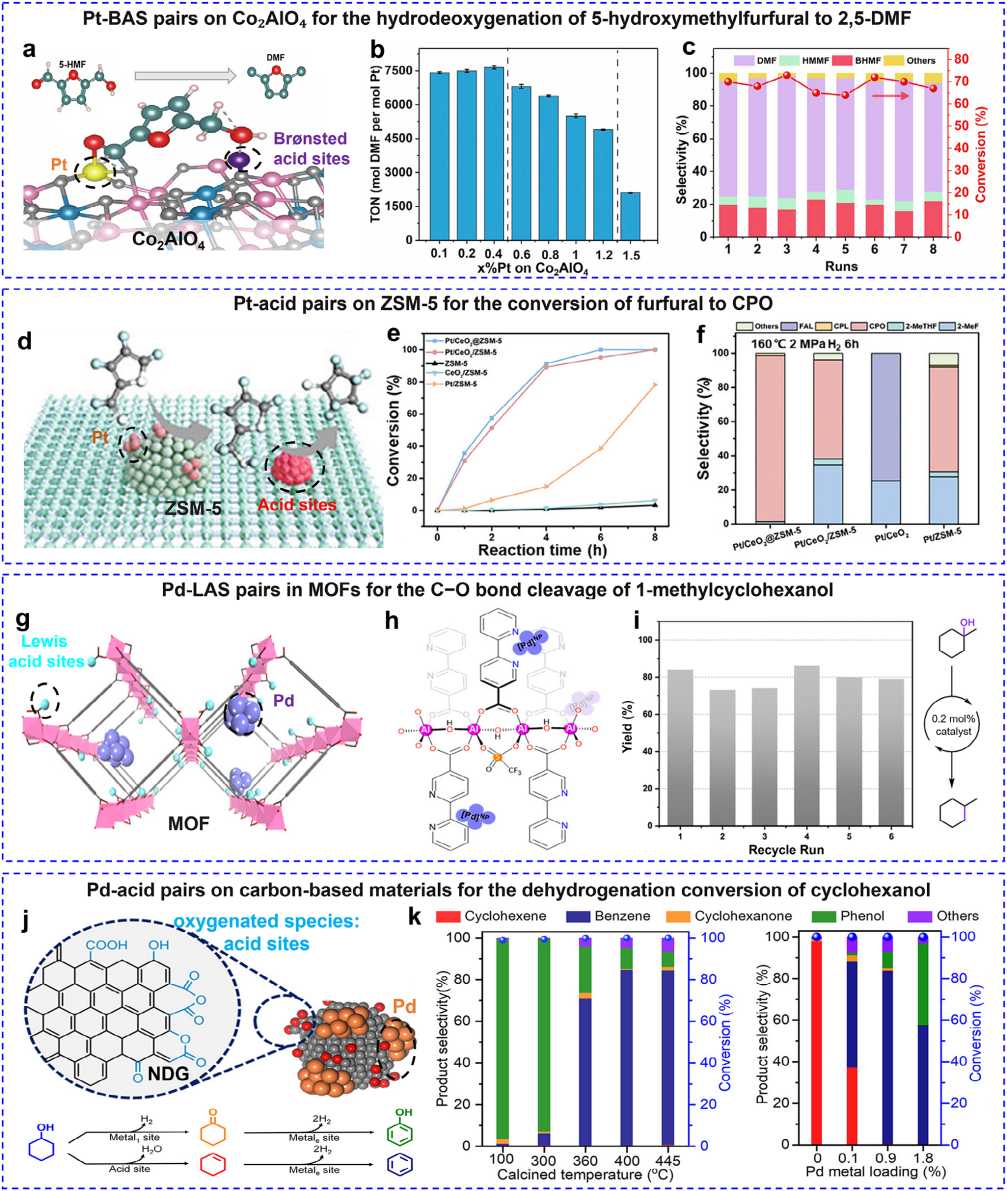
The development of some synthesis approaches for achieving neighboring active motifs with metal‐acid (Brønsted/Lewis) pairs in different support materials. a) The Pt‐BAS pairs on Co₂AlO₄ support are used for the synergistic catalytic hydrodeoxygenation of 5‐hydroxymethylfurfural to 2,5‐DMF. b) TOF values for Pt/Co_2_AlO_4_ catalysts with various Pt loadings. c) Reusability tests on the 0.4% Pt/Co_2_AlO_4_ catalyst. a–c) Reproduced with permission.^[^ ^113^ ^]^ Copyright 2022, Elsevier Inc. d) Schematic illustration for sequential furfural hydrogenation over Pt/CeO_2_@ZSM‐5, Pt/CeO_2_/ZSM‐5, and Pt/CeO_2_. e) Furfural hydrogenation conversion as a function of reaction time at 160 °C under 2 MPa H_2_ and f) corresponding product distribution in 6 h. d–f) Reproduced with permission.^[^ ^115^ ^]^ Copyright 2024, Wiley‐VCH GmbH. g, h) LASs and Pd NPs in MOFs. i) Recycle experiments for Pd/NUS‐SO_3_H‐catalyzed C−O bond cleavage of 1‐methylcyclohexanol. g–i) Reproduced with permission.^[^ ^118^ ^]^ Copyright 2020, American Chemical Society. j) Pd‐acid pairs on C‐based materials used for the synergistic catalytic dehydrogenation conversion of cyclohexanol. k) Cyclohexanol conversion and product selectivity over the Pd/NDO catalysts with different acid site densities and different metal loadings. Conditions: catalyst (150 mg), cyclohexanol 1.7 kPa, weight hourly space velocity (WHSV) of 0.6 g_cyclohexanol_ g_cat._^−1^ h^−1^, N_2_ 20 mL min^−1^ (STP), 300 °C. j–k) Reproduced with permission.^[^ ^141^ ^]^ Copyright 2024, American Chemical Society.

Various oxide supports, including Al₂O₃, MgO, Nb₂O₅, TiO₂, ZrO_2_, and SnO₂, have been found to offer metal‐acid pairs where the lattice oxygen atoms can stabilize active motifs for C−O and C−C bond activation. For example, Meng et al. developed a Mo₁Al/MgO catalyst featuring atomically dispersed Mo centers and Al LASs on a MgO substrate for efficient depolymerization of Eucalyptus lignin. The catalyst uses Mo sites for *β*‐aryl ether bond cleavage and Al acid sites to activate lignin and stabilize intermediates. DFT and experimental findings revealed the synergy between Mo_1_─O_5_ single atoms and Al LASs in the presence of methanol, resulting in the production of aromatic monomers with a high selectivity of 92%.^[^ ^111^ ^]^ Leveraging the strong interaction between Ru and Nb_2_O_5_, Ru/Nb₂O₅ catalysts achieved 75–85% yields of arenes from aromatic plastic waste hydrogenolysis. The dispersed Ru clusters near the metallic zero‐valence state prevent aromatic ring hydrogenation, while NbO_x_ species activate C−O and C−C bonds via Lewis and Brønsted acidity.^[^ ^112^ ^]^ Single‐atom Pt on Co₂AlO₄ spinel enhanced Brønsted acidity at adjacent Co−O(H)−Al sites (**Figure** **9**a), achieving 99% yield and high turnover frequency (2553 h⁻¹) for hydrodeoxygenation of 5‐hydroxymethylfurfural to 2,5‐dimethylfuran (Figure 9b,c). This synergistic interaction between Pt atoms and acid sites enables selective activation of C═O bonds and efficient C─O cleavage.^[^ ^113^ ^]^ Qin et al. developed a rationally designed bifunctional catalyst featuring core‐shell structured ZrO_2_ and Co nanoparticles. In this system, the redox‐active Co sites facilitate the hydrogenation of nitroarenes, while the Lewis basic ZrO_2_ promotes the N‐formylation of aryl amines with CO_2_. This dual functionality is enabled by their optimized electronic configurations and efficient mass transport of reactants and intermediates.^[^ ^17^ ^]^ Noh et al. explored Cu/SiO_2_ catalysts modified with LASs (Ti, Zr, Hf, Nb, Ta) using surface organometallic chemistry for CO_2_ hydrogenation. Methanol formation rates correlated with Lewis acid strength, assessed through pyridine adsorption enthalpies and methoxy ^13^C chemical shifts. Stronger Lewis acids stabilized intermediates near Cu, boosting CH_3_OH formation. The study revealed that π‐bonding in M─OCH_3_ influences reactivity, offering insights for designing efficient CO_2_ hydrogenation catalysts.^[^ ^114^ ^]^ Recent studies highlight that incorporating ordered mesopores into metal oxides significantly enhances acidic site accessibility due to abundant V_O_, thereby boosting the synergistic catalytic activity of metal‐acid pairs.^[^ ^139^, ^140^ ^]^ Xiao et al. showcased the efficacy of 3D ordered mesoporous SnO_2_ single crystals as a superior substrate for Pd nanoparticle immobilization, facilitating the stepwise hydrogenation of 4‐nitrophenylacetylene to yield 4‐nitrostyrene, followed by 4‐nitroethylbenzene, and culminating in 4‐aminoethylbenzene. This transformation is driven by optimal electron transfer between Pd and SnO_2_, highlighting their synergistic interaction.^[^ ^139^ ^]^ Building on this, the team further demonstrated that integrating Pt nanoparticles with highly ordered mesoporous SnO_2_ single crystals results in highly effective bifunctional catalysts. These catalysts enable the tandem conversion of bio‐furans into BTX aromatics (benzene, toluene, and *p*‐xylene), achieving an exceptional *p*‐xylene yield of 96.1% through the aromatization of 2,5‐dimethylfuran with acrylic acid.^[^ ^140^ ^]^

Zeolites, with their highly crystalline structure of interconnected SiO₄ tetrahedra, are ideal for embedding metal‐acid pairs. Their channels and pores enable precise control over catalytic cluster size and distribution, preventing aggregation and enhancing activity. Substitution of Si⁴⁺ with Al^3^⁺ introduces negative charges, neutralized by protons to form BASs, essential for hydrocarbon and oxygenate transformations in petroleum refining and petrochemicals.^[^ ^142^ ^]^ TMs can be incorporated into the framework, leading to the formation of LASs. Adjusting the Si/T ratio (T = Al, Ga, Sn^[^ ^143^ ^]^) allows precise control over acid site density, optimizing catalytic performance. A “confined auto‐redox” strategy produced a Pt/CeO₂@ZSM‐5 catalyst for furfural conversion to cyclopentanone (CPO) (Figure 9d). CeO₂ regulators enhanced Pt‐acid interactions, achieving >99% conversion and 97.2% selectivity at 160 °C (Figure 9e,f).^[^ ^115^ ^]^ A template‐guided method synthesized sub‐nanometric Pt clusters (0.89–1.22 nm) encapsulated in beta‐zeolite via electrostatic interactions between PtCl₆^2^⁻ and charged templates. Reduction in H₂ yielded Pt@beta, whose electron‐withdrawing framework promoted hydrogen transfer, enhancing hydrodeoxygenation performance.^[^ ^144^ ^]^ Additionally, Pt nanoparticles encapsulated in silicalite‐1 (Pt@S‐1) achieved 89.5% naphtha yield with 96.8% selectivity for C₅‐C₉ hydrocarbons at 250 °C. These examples highlight the synergistic effects of zeolite acidity and confined metal particles in catalytic applications.^[^ ^116^ ^]^

MOFs with tunable organic linkers enable the integration of metal‐acid pairs to enhance catalytic performance. Functionalizing MOFs with acidic groups increases acid site density and strength. A 2D MOF catalyst (Pd/NUS‐SO_3_H) was synthesized via diazotization with 4‐aminobenzenesulfonic acid and PdCl_2_ impregnation. This catalyst, with closely spaced Pd, SO_3_H, and Zr^4+^, achieved superior yields of 2,5‐dimethylfuran (2,5‐DMF) from saccharides compared to previously reported catalysts. The reduced diffusion distances in 2D MOFs amplify the synergistic effects of acid and hydrogenation catalysis for 2,5‐DMF production.^[^ ^117^ ^]^ Another multistep strategy was also developed to create LASs and Pd nanoparticle catalysts in a mixed‐ligand MOF comprising (Al‐OH)_n_ nodes (Figure 9g,h). Triflation of Al_2_(OH)(OH_2_) sites formed strong Lewis acidic centers, while Pd(MeCN)_2_Cl_2_ coordination and H_2_ reduction generated Pd nanoparticles. These Pd‐LAS pairs demonstrated high catalytic efficiency and reusability for C−O bond cleavage, highlighting their potential for tandem catalysis (Figure 9i).^[^ ^118^ ^]^

Carbon‐based materials, widely recognized as inert supports for metal dispersion, can be functionalized with oxygen‐containing groups, which introduces acidity. Zhang et al. demonstrated that the calcination of nanodiamond@graphene (NDG) particles introduces acid and Pd sites, notably altering the reaction pathway for cyclohexanol conversion (Figure 9j). This process enriches the NDG surface with oxygenated species, such as carboxylic anhydrides and phenolics, which facilitate dehydration reactions. By adjusting the density of acidic and metal sites on Pd/NDG catalysts, selectivity toward dehydration, dehydrogenation, or combined products can be fine‐tuned (Figure 9k).^[^ ^141^ ^]^ Additionally, atomically dispersed Al/Ga−O/N‐C structures can be incorporated into carbon‐based materials to modulate Lewis acidity and enhance catalytic synergy.^[^ ^145^, ^146^ ^]^ Wang et al. developed a carbon‐based catalyst with atomically dispersed Fe‐Al dual sites, achieving 97% conversion and 91% selectivity in olefin oxidative carboxylation. The enhanced performance stems from synergistic effects: the Lewis acidic Al sites promote intermediate (styrene oxide) consumption, significantly improving oxidation rates at Fe sites and shifting the equilibrium toward epoxidation.^[^ ^119^ ^]^

Metal‐acid pairs are crucial for biomass conversion, refining, and hydrocarbon transformation. Future advancements focus on precise design via synthesis techniques, computational modeling, and exploring novel materials to enhance efficiency, selectivity, and sustainability in catalysis.

### 3.2 Metal‐Base Pairs

Metal‐base pairs also play a crucial role in heterogeneous catalysis by enabling synergistic interactions between nucleophilic and electrophilic species. Unlike acid catalysts, base catalysts activate substrates through electron donation, facilitating nucleophilic substitution reactions. Basicity in traditional heterogeneous catalysts is typically derived from alkali or alkaline earth metals and N‐containing ligands or functional groups. In metal‐base pairs, the basic site can act analogously to an acid site, which promotes reactions through alternative pathways and supporting complex mechanisms. This pairing allows simultaneous activation of nucleophiles and electrophiles, enhancing reactivity and selectivity. Base sites also serve as proton shuttles, enabling cascade reactions such as positional isomerizations. Research on metal‐base catalysts often focuses on Lewis acid‐base (LAS‐LBS) pairs, with the metal sites typically acting as LASs. Based on the matching of steric hindrance or orbital energy, acid‐base pairs can be categorized into classical LAS‐LBS pairs and FLPs. Classical LAS‐LBS pairs can form donor‐acceptor bonds. In contrast, frustrated Lewis pairs, because of steric hindrance, prevent the formation of such bonds.

The alkalinity of metal oxides is influenced by electronegativity and the nature of M─O bonds in surface hydroxyl groups. Strong M─O bonds dissociate H⁺ and exhibit acidic properties, whereas weak M─O bonds dissociate OH⁻, imparting alkalinity. These properties make metal‐base pairs highly versatile in catalysis. Metal oxides are widely utilized for constructing such active sites, offering surfaces with LASs (unsaturated cationic metal centers) and LBSs (anionic oxygen centers) which can activate C─H bonds in alkanes and alcohols.^[^ ^147^ ^]^ Jaegers et al. demonstrated the high reactivity of LAS‐LBS pairs at ZrO_2_ powders for the dehydrogenation of C_2_‐C_4_ alkanes. The ZrO_2_ catalysts activate C─H bonds in oxygenates via heterolytic routes on exposed Zr^δ+^─O^δ−^ site pairs, resulting in bounding anionic alkyl species and cationic hydrogen species. Further research focusing on the controlled formation of active and stable LAS‐LBS pairs in ZrO_2_ or other metal oxide materials holds significant potential in terms of replacing toxic and expensive metals with more abundant alternatives.^[^ ^148^ ^]^

However, conventional LAS‐LBS pairs suffer from inherent incompatibility between adjacent acidic and basic sites, which typically leads to the formation of thermodynamically stable adducts with diminished catalytic reactivities. Research in recent years has found that when LASs and LBSs are sterically hindered and located in proximity, FLPs can be formed. This innovative approach effectively prevents self‐deactivation caused by strong chemical interactions while preserving both the stability and accessibility of active catalytic centers, which are effective in the activation of small molecules in reactions (e.g., H_2_, CO_2_, H_2_O, etc.). They generally feature synergistic collaboration during catalysis, where the LAS accept electrons and Lewis base sites donate electrons to reaction substrate(s).^[^ ^149^ ^]^ However, traditional molecular‐type FLP molecular catalytic systems face challenges in product purification and catalyst recovery, driving more interest in using recyclable heterogeneous catalysts.^[^ ^62^, ^125^, ^150^ ^]^ FLPs can be constructed over the surface of defective metal oxides and hydroxides such as CeO_2_, Al_2_O_3_, TiO_2_, Nb_2_O_5_, and In_2_O_3–x_(OH)_y_ through doping, defect‐engineering strategy, or functional group modification, which is efficient in promoting H_2_ dissociation, H_2_O splitting, and CO_2_ reduction.^[^ ^62^, ^64^, ^151^ ^]^

**Figure 10:**
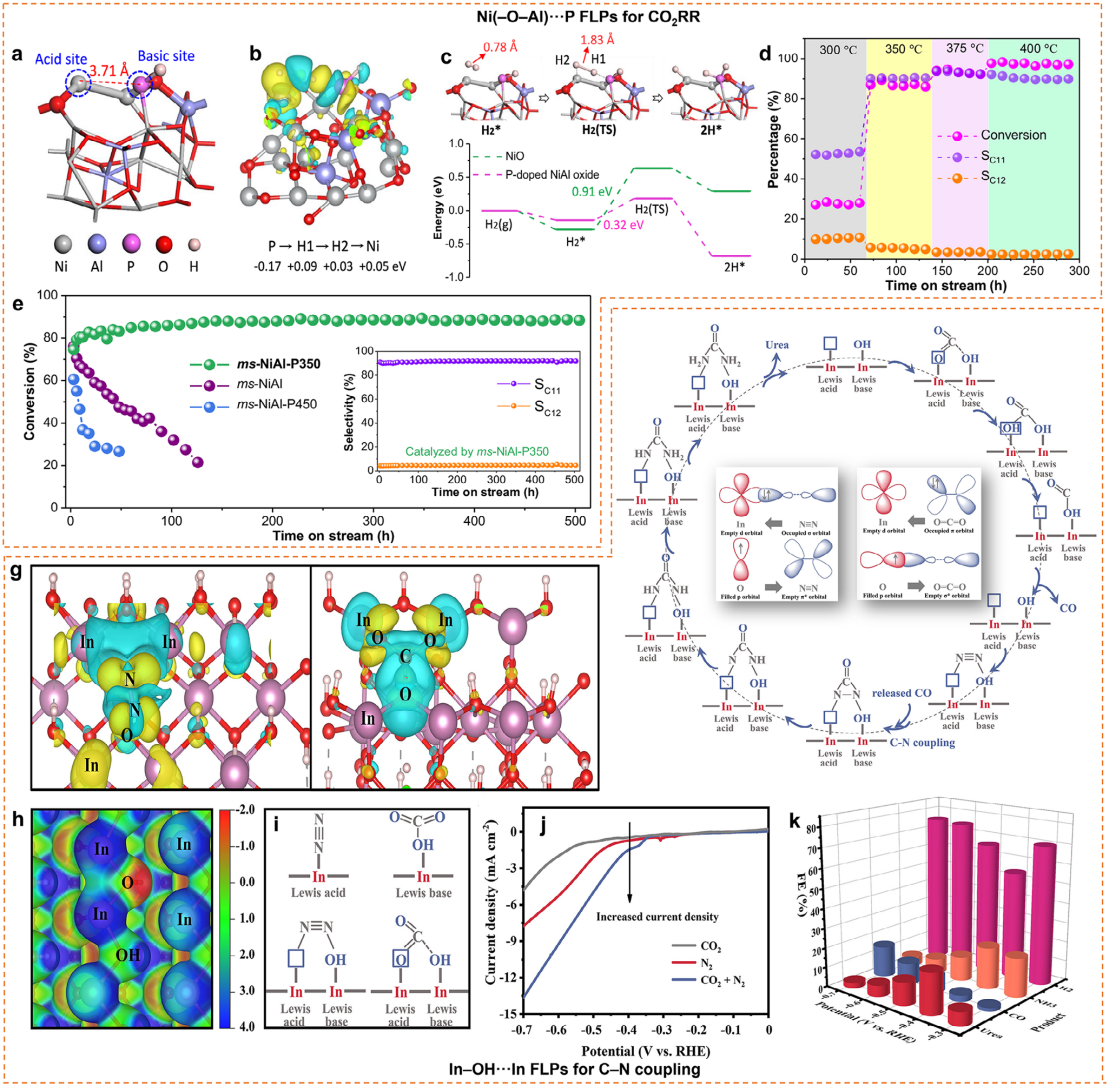
Synthesis development for achieving neighboring active motifs with metal‐base pairs (FLPs). a) The proposed model of Ni(─O─Al)···P FLPs in P‐doped NiAl oxide. b) The differential charge density distribution of H_2_ activation on the Ni(─O─Al)···P FLPs (yellow isosurface means electron accumulation, blue isosurface means electron depletion). c) The proposed H_2_ activation process on P‐doped NiAl oxide and the corresponding energy change profiles. d) Temperature screening for hydrotreating of methyl laurate over *ms*‐NiAl‐P350. Reaction conditions: P_H2_  =  3.0 MPa, WHSV  =  28.3 h^−1^. e) Methyl laurate conversions as a function of time over *ms*‐NiAl‐P350 and the reference catalysts. The inset shows the selectivity of C_11_H_24_ and C_12_H_26_ over *ms‐*NiAl‐P350. Reaction conditions: *T*  =  350 °C, *P*_H2_  =  3.0 MPa, WHSV  =  28.3 h^−1^. a–e) Reproduced with permission.^[^ ^120^ ^]^ Copyright 2024, The Author(s), published by Nature Publishing Group. f) Schematic illustration of the electrocatalytic co‐activation of N_2_ and CO_2_ into urea over artificial FLPs of InOOH‐100 catalysts. g) The charge density difference of N_2_ and CO_2_ adsorbed on FLPs sites; the yellow and cyan colors indicate electron accumulation and depletion, respectively, with an isosurface value of 0.013 e Å^−3^. h) The electrostatic potential surface of InOOH (001), the electron‐density isosurface is plotted at 0.02 eÅbohr^−3,^ and the color bar represents the electrostatic potential scale. i) Schematic illustration of N_2_ and CO_2_ molecules adsorbed on independent LAS, LBS, and FLP sites. j) The linear sweep voltammograms (LSV) of InOOH‐100 catalysts in CO_2_, N_2_ and CO_2_ + N_2_ saturated electrolyte. k) The corresponding product distribution of H_2_, CO, NH_3_, and urea with N_2_ and CO_2_ as the feeding gas at various potentials for InOOH‐100 catalysts. f–k) Reproduced with permission.^[^ ^121^ ^]^ Copyright 2021, Elsevier Inc.

Layered double hydroxides (LDHs), or hydrotalcite‐like compounds, are 2D anionic clays with positively charged metal hydroxide layers and interlayer anions or water molecules. Upon calcination, LDHs like MgAl‐LDHs convert into mixed metal oxides (MMOs) with a range of surface basic sites: weak (OH⁻), medium (Mg−O Lewis acid‐base pairs), and strong (low‐coordinated O^2^⁻ ions).^[^ ^152^ ^]^ Xiao et al. designed bifunctional Ni_x_Mg_y_‐MMO catalysts by varying Ni/Mg ratios in NiMgAl‐LDH precursors. The Ni₃Mg₁‐MMO catalyst excelled in methanol aqueous‐phase reforming, achieving a high hydrogen production rate (167.2 µmol_H₂_ g_cat_^−1^ s^−1^) and low CO selectivity (1.9%) at 240 °C. This performance arose from the synergy between the Ni^0^ sites for dehydrogenation and Mg−O sites for water activation and hydroxyl formation, promoting the water‐gas shift reaction.^[^ ^153^ ^]^ Additionally, Li et al. reported a scalable P‐doped NiAl‐oxide catalyst with FLPs, where the P sites (as LBSs) synergize with Ni(−O−Al) (as LASs) (**Figure** **10**a). This unique structure demonstrated outstanding efficiency in sulfur‐free green diesel production, with the NiAl‐oxide‐P interaction being key to its catalytic performance (Figure 10b–e). The structural flexibility of LDH precursors offers broad potential for FLP‐type catalysts beyond green diesel synthesis.^[^ ^120^ ^]^ Yuan et al. developed FLPs in InOOH‐100, where unsaturated In sites and neighboring hydroxyl groups serve as LASs and LBSs, respectively (Figure 10f–k). They form rice‐like nanoparticles that produce a urea yield of 6.85 mmol h⁻¹ g⁻¹ and FE of 20.97% at −0.4 V versus RHE (Figure 10j,k). The electron‐deficient LASs chemisorb N₂, while electron‐rich LBSs activate CO₂, enabling targeted interactions and generating intermediates for urea synthesis (Figure 10g‐i). FLPs facilitate ∗N = N∗ and CO coupling into ∗NCON∗ precursors, driving efficient C─N bond formation (Figure 10f). The synergistic LAS‐LBS activity enables remarkable performance in urea synthesis.^[^ ^121^ ^]^ Liang et al. synthesized a ZnIn₂S₄/In(OH)_3‐x_ heterojunction for visible‐light‐driven CO₂ reduction. Here, ZnIn₂S₄ harvested light while hydroxyl‐deficient vacancies in In(OH)_3‐x_ acted as LAS and adjacent hydroxyl groups as LBS, forming FLPs that activated CO₂ for reduction.^[^ ^122^ ^]^ These studies highlight the versatility of LDH‐derived materials in catalysis.

**Figure 11:**
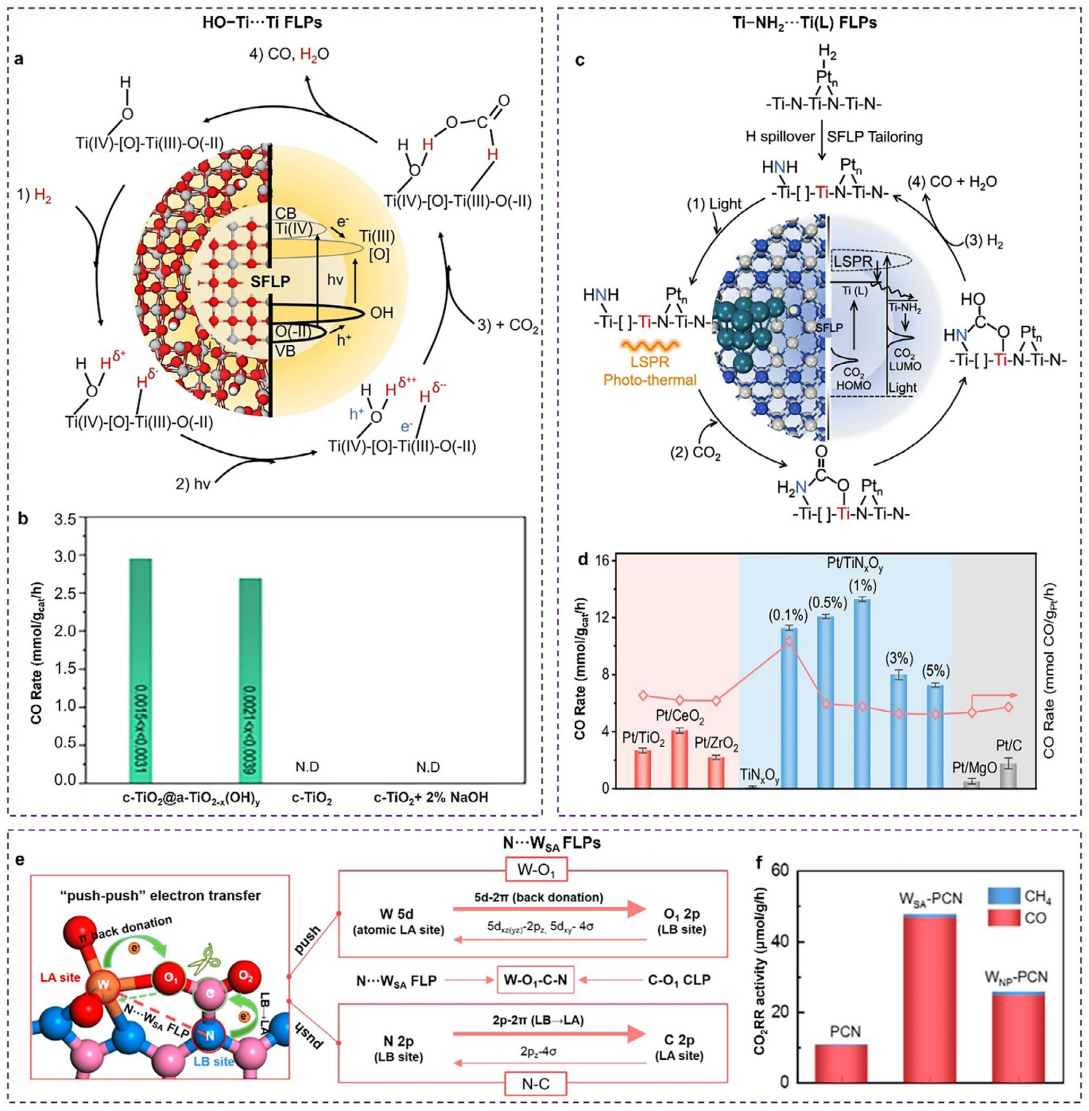
Heterogeneous FLP catalysts used for CO_2_ photoreduction reactions. a) Depiction of the central role played by the physicochemical properties in the catalytic hydrogenation of CO_2_‐to‐CO by HO‐Ti···Ti FLPs. b) CO production rates of c‐TiO_2_@a‐TiO_2‐x_(OH)_y_, pristine c‐TiO_2_ and 2% Na/c‐TiO_2_ under visible light (2.8 W cm^−2^). a,b) Reproduced with permission.^[^ ^123^ ^]^ Copyright 2022, The Author(s), published by Nature Publishing Group. c) Depiction of the central role played by the physicochemical properties in the catalytic hydrogenation of CO_2_‐to‐CO by Ti−NH_2_···Ti(L) FLPs. d) Photo‐thermal catalytic performance of all tested samples. c,d) Reproduced with permission.^[^ ^125^ ^]^ Copyright 2024, The Author(s), published by Nature Publishing Group. e) Schematic diagram of CO_2_ adsorption on N···W_SA_ FLP. f) Average reaction rate of main CO_2_RR products. e,f) Reproduced with permission.^[^ ^124^ ^]^ Copyright 2025, American Chemical Society.

Traditional gas‐phase heterogeneous surface FLP catalysts primarily involve metal oxide‐hydroxides (MOH···M). Li et al. formed a core‐shell c‐TiO₂@a‐TiO_2‐x_(OH)_y_ heterostructure through a solid‐state reduction reaction between sodium and TiO₂, comprised of HO─Ti···Ti surface FLPs (**Figure** **11**a). The engineered TiO_2_ demonstrates greatly enhanced photocatalytic activity. These surface FLPs heterolytically dissociate H_2_ at room temperature to form charge‐balancing protonated hydroxyl groups and hydrides at unsaturated titanium surface sites, achieving a CO_2_‐to‐CO conversion rate of 5.3 mmol g_cat_^−1^ h^−1^ under solar irradiation (Figure 11b). Additionally, the turnover frequency reached 592 h^−1^, showcasing the effectiveness of surface FLPs in driving multi‐electron reductions.^[^ ^123^ ^]^ To enhance catalytic performance, a functional group modification strategy has been developed to improve the process performance metrics in replacing the Lewis basic MOH with a stronger alternative; an intriguing example being the amine MNH_2_ in metal nitrides. Zou et al. introduced a pioneering photoactive surface FLPs, achieved by integrating platinum nanoparticles with titanium nitride (TiN_x_O_y_) (Figure 11c). Under CO_2_ hydrogenation conditions, hydrogen spillover from Pt generates low‐valent titanium (Ti(L)) and adjacent Ti−NH_2_ groups, forming the Ti−NH_2_···Ti(L) FLP on the surface. This catalyst leverages the strong plasmonic properties of TiN_x_O_y_ for light‐driven CO_2_ reduction, facilitating the formation of a key carbamate intermediate that converts into CO (Figure 11d). The study highlights the potential of metal (oxy)nitrides in advancing FLP‐based catalytic systems for sustainable applications.^[^ ^125^ ^]^ Xu et al. constructed atomic W‐based FLP sites on polymeric carbon nitride (PCN) for photocatalytic CO₂ conversion (Figure 11e). In the system, W single atoms serve as LASs, while adjacent N atoms act as LBSs, enabling CO₂ activation via a unique W─O─C─N structure with significant *d*–*p* orbital interactions. A “push‐push” electron transfer mechanism, involving π back‐donation from W and electron transfer from N to CO₂, effectively cleaves the C═O bond, which notably enhances the CO₂‐to‐CO conversion efficiency (Figure 11f).^[^ ^124^ ^]^ These findings showcase the potential of FLPs for advanced catalytic applications.

**Figure 12:**
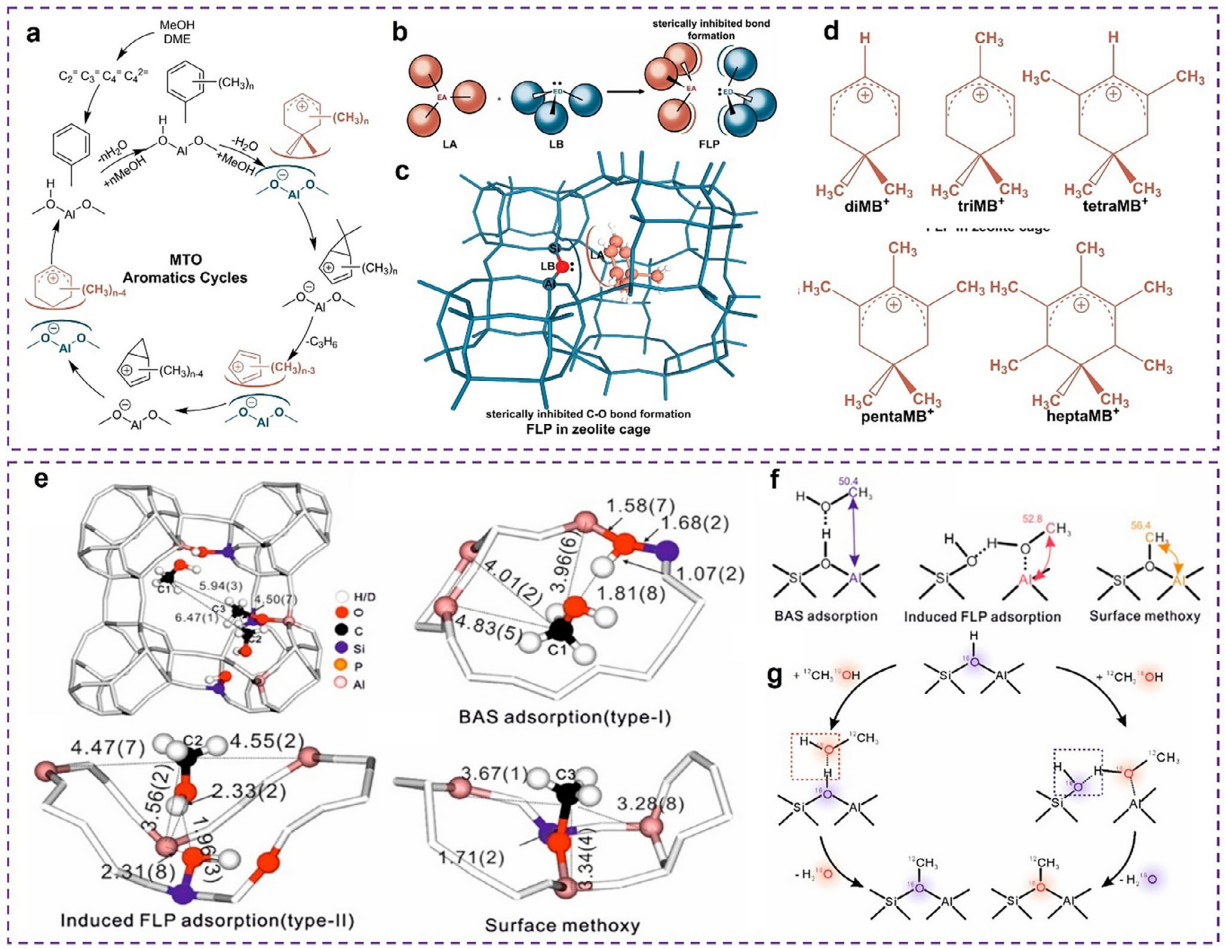
FLPs in zeolites. a) Reaction mechanism of aromatics cycles in the zeolite‐catalyzed MTO reaction. b) Traditional FLP‐impeded dative bonding between LAS and LBS. c) FLP‐impeded covalent bonding between Si─O─Al^−^ (LBS) and PMB^+^ (LAS) in the zeolite cage. d) Five common PMB^+^ that are discussed in this work. EA: electron acceptor, ED: electron donor. a–d) Reproduced with permission.^[^ ^154^ ^]^ Copyright 2022, Wiley‐VCH GmbH. e) Crystallographic model based on the combined Rietveld refinement of SXRD and neutron diffraction of methanol adsorbed on BAS, the framework Al site, and the formed surface methoxy species. f) The different types of adsorbed methanol on the surface of zeolites. g) The mechanism of methanol‐induced FLP formation. e‐g) Reproduced with permission.^[^ ^155^ ^]^ Copyright 2021, American Chemical Society.

Despite progress in heterogeneous FLP catalysis, challenges such as random site distribution, agglomeration, formation of classic Lewis pairs, and difficulty isolating FLP interactions hinder optimal site utilization. The concept of FLP is extended to zeolite catalytic systems, which provides a new way to solve the above problems. Chen et al. discussed the catalytic properties of deprotonated zeolites SAPO‐34 and SSZ‐13 in facilitating hydride and C─H bond activation through an FLP mechanism (**Figure** **12**a–d). Specifically, the positively charged atom of polymethylbenzenium interacts with Si─O─Al sites to effectively dissociate H_2_ and activate alkanes, particularly propane to propene. The study finds that the carbocations generated during the methanol conversion process in the cage‐like SSZ‐13 and SAPO‐34 zeolites serve as natural LAS due to their electron‐deficient nature. Their formation is accompanied by deprotonated BASs (Si─O─Al─), with the negatively charged oxygen atom acting as an electron‐rich LBS (Figure 12b,c). Importantly, the direct bonding between the carbocations and Si─O─Al─ is hindered by the confinement effect of the zeolitic pores, resulting in a sterically hindered LAS‐LBS pair. Experimental results show enhanced catalytic activity of these FLPs compared to classical FLPs, with conversion efficiency linked to the Si/(P+Al) ratio. The study emphasized the potential for improving alkane dehydrogenation processes using these zeolite‐based FLPs in catalytic applications.^[^ ^154^ ^]^ Li et al. provided the first experimental evidence of induced active sites in silicoaluminophosphate (SAPO) zeolites influenced by polar adsorbates (Figure 12e–g), challenging the notion of fixed active sites. Their findings show that polar adsorbates transiently create FLPs, with a three‐coordinated framework Al serving as an LAS and SiO(H) as an LBS. Notably, the BASs (Al(OH)Si) in SAPO can transform into FLP structures upon binding strong polar adsorbates.^[^ ^155^ ^]^ For instance, acetone adsorption on BASs thermally converts into an FLP adsorption mode in SAPO‐34.^[^ ^156^ ^]^ Furthermore, designing atomically precise FLP catalysts provides valuable insights into catalytic mechanisms and industrial applications. Leung et al. demonstrated that atomically dispersed Ru species confined in 13X zeolite cavities exhibit exceptional catalytic activity (>4000 h⁻¹) for NH₃ decomposition. The Ru^δ⁺^···O^δ⁻^ FLP structure facilitates heterolytic N─H bond cleavage, in contrast to the homolytic cleavage typical of metal nanoparticles, as confirmed by advanced structural characterization techniques such as probe‐assisted SXRD in combination with the Rietveld refinement.^[^ ^157^ ^]^

The integration of FLPs into heterogeneous catalysts has opened transformative pathways for small molecule activation, offering solutions for energy conversion and sustainable chemical synthesis. Advances in structural design, doping, and defect engineering of materials such as metal oxides, LDHs, and MOFs continue to enhance their catalytic potential. By expanding the diversity and scalability of FLP‐based systems, future research can address critical challenges in green chemistry and industrial catalysis.

### 3.3 Metal–Metal Pairs

Metal‐metal pairs, consisting of either homonuclear or heteronuclear metals positioned closely together, enable synergistic catalysis and can greatly enhance catalytic reactivity. TMs, due to their unique redox properties, are ideal candidates, offering extensively tunable versatility for the construction of such metal‐metal pairs. While similar to SACs, metal‐metal pairs pose unique challenges, including precise dispersion on support surfaces, prevention of aggregation, and optimization of interatomic distances and coordination environments. Addressing these complexities through innovative synthesis techniques has resulted in advanced catalysts with a wide range of catalytic applications. The uniform distribution of homonuclear and heteronuclear metal‐metal pairs is intricately linked to the synthesis method. Techniques such as atomic layer deposition (ALD), high‐temperature pyrolysis, and wet chemical methods offer diverse strategies to incorporate metal sites onto supports.

ALD is a precise technique for synthesizing metal‐metal pairs by leveraging sequential, self‐limiting reactions, enabling atomic‐level control.^[^ ^158^ ^]^ Adjustments to ALD cycles fine‐tune metal loading and interatomic distances, improving catalytic performance. A typical two‐step ALD process deposits a metal‐organic precursor onto defect sites at elevated temperatures, followed by a second precursor to form controlled metal‐metal interactions. Cyclopentadienyl‐based metal complexes are commonly used for their stability and controlled metal release, which prevents aggregation.^[^ ^159^ ^]^ For instance, ozone exposure during synthesis oxidizes metal sites (such as Pt‐Ni and Ru‐Pt), enhancing their oxidation state for stability and catalytic efficiency.^[^ ^131^, ^133^ ^]^

High‐temperature pyrolysis is another effective approach for forming metal‐metal pairs, converting metal‐organic complexes or frameworks into metal‐nitrogen‐carbon (M─N─C) catalysts under controlled conditions. Organic components in the precursors transform into defect‐rich carbon matrices that trap metal atoms and form neighboring pairs such as Fe‐Mn,^[^ ^135^ ^]^ Pd–Mn,^[^ ^134^ ^]^ and Co–Ni.^[^ ^136^ ^]^ Notably, the in situ‐generated carbon can act as a reducing agent, enabling the formation of low‐oxidation‐state metal pairs with outstanding catalytic properties.

Wet chemical methods, including electrochemical and photochemical approaches^[^ ^160^ ^]^ are increasingly favored for synthesizing metal‐metal pairs under mild conditions. These techniques eliminate the need for high temperatures, reducing the risk of site aggregation and ensuring structural integrity. Additionally, their scalability makes them attractive for commercial applications. By preserving active motifs and enabling precise control over metal interactions, these methods complement ALD and pyrolysis for catalyst synthesis.

#### 3.3.1 Homonuclear Metal–Metal Pairs

Homonuclear metal‐metal pairs, particularly those involving Fe₂, Cu₂, Pt₂, and Pd₂, have captivated extensive research interest. Their allure lies in the enhanced catalytic activity achieved through synergistic catalysis. These catalysts have been the subject of in‐depth exploration across a diverse range of reactions, such as ORR, CO_2_RR, N₂O reduction reaction (N₂ORR), and so forth. Moreover, the development of dual‐site catalysts inspired by biological enzymes has emerged as a burgeoning research direction. This convergence of research efforts not only showcases the potential of homonuclear metal‐metal pairs in revolutionizing catalytic processes but also underscores the innovative approach of drawing inspiration from nature to drive advancements in the field of catalysis.

##### ORR

**Figure 13:**
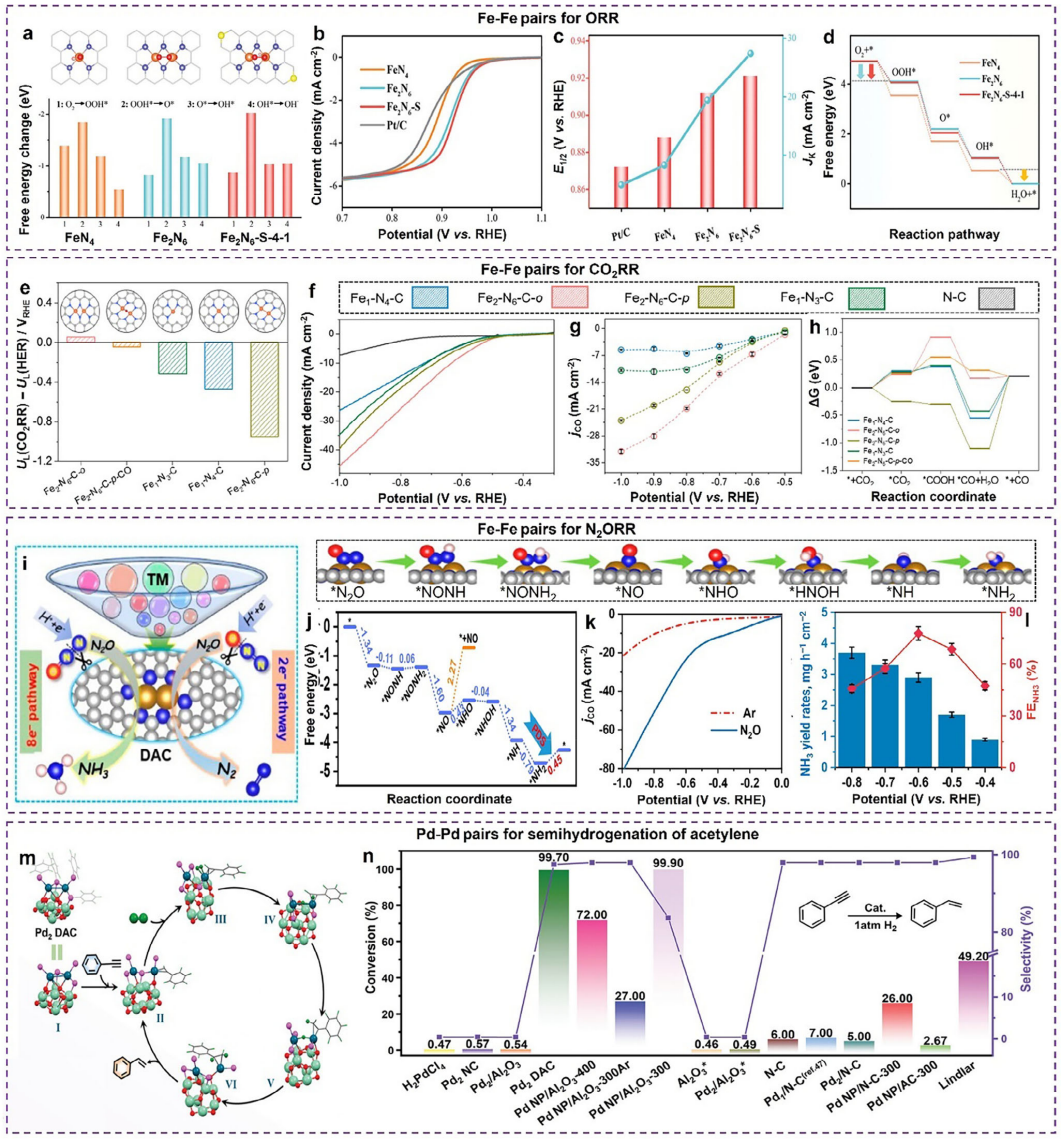
Homonuclear metal‐metal pairs. a) Free energy changes for each reaction step of ORR. Electrocatalytic performance evaluation of samples in 0.1 m KOH. b) LSV curves. c) Comparison of *E*_1/2_ and kinetic current densities at 0.88 V versus RHE. d) Free energy step diagrams of ORR. a–d) Reproduced with permission.^[^ ^127^ ^]^ Copyright 2024, Wiley‐VCH GmbH. e) Difference in theoretical limiting potential for CO_2_RR and HER for Fe_1_─N_4_─C, Fe_2_─N_6_─C‐*p*‐CO, Fe_1_─N_3_‐C, Fe_2_─N_6_─C‐*o*, and Fe_2_─N_6_─C‐*p*. f, g) The LSV curves f) and CO partial current density (g) in 0.5 M CO_2_/Ar‐saturated KHCO_3_ electrolyte. h) Free energy diagram of CO_2_RR. e–h) Reproduced with permission.^[^ ^10^ ^]^ Copyright 2022. American Chemical Society. i) Schematic illustration of the N_2_ORR over DAC: through the 2e pathway toward N_2_ or 8e pathway toward NH_3_. j) Free energy diagrams and key intermediate configurations for N_2_ORR on Fe_2_ DAC. k) LSV curves of Fe_2_@NG DAC in Ar/N_2_O‐saturated 0.5 M Na_2_SO_4_. l) Obtained NH_3_ yield rates and FE_NH3_ on Fe_2_@NG. i–l) Reproduced with permission.^[^ ^129^ ^]^ Copyright 2024, American Chemical Society. m) Mechanism of the Pd_2_ DAC catalyzing the semi‐hydrogenation of phenylacetylene. n) Comparison of the catalytic activities of different catalysts in the semi‐hydrogenation of acetylene in the liquid phase under the same reaction conditions. (Reaction conditions: substrate, 1.0 mmol; amount of metal Pd, 2.8 × 10^−4^ mmol; C_2_H_5_OH, 0.55 mL; H_2_ balloon; 30 °C; 3 h). m,n) Reproduced with permission.^[^ ^163^ ^]^ Copyright 2024, Wiley‐VCH GmbH.

Building on the exploration of Fe₂‐based catalysts for oxygen‐related reactions, the O_2_ adsorption and substrate interactions have been modulated, resulting in improved catalytic activity of ORR. Yan et al. developed Fe₂ catalysts with N₂─Fe─N₂─Fe─N₂ motifs using a sublimation transformation synthesis strategy. The catalyst features a Fe···Fe distance of 0.30 nm, which enhances O₂ adsorption and protonation, resulting in superior ORR performance with a half‐wave potential of 0.90 V (versus RHE) and a kinetic current density of 20.99 mA cm^−2^ at 0.85 V, showing minimal activity decay after 10 000 cycles. The lengthened Fe···Fe distance (0.30 nm) compared to catalysts with shorter Fe···Fe distances (0.25 nm) promotes easier protonation of *O₂ to *OOH and prevents O₂ poisoning, and improves ORR activity.^[^ ^126^ ^]^ Similarly, Ye et al. developed a method to precisely control the number of Fe atoms in clusters anchored on NCs, enabling atomic‐level investigation of ORR catalyst design. By adjusting the number of clustered Fe atoms, they optimized O₂ adsorption modes from superoxo‐like to peroxo‐like, greatly enhancing acidic ORR activity. The catalyst achieved a half‐wave potential of 0.78 V (vs RHE) with only a −20 mV shift after 20 000 cycles in 0.5 m H₂SO₄, comparable to commercial Pt/C.^[^ ^161^ ^]^ Biological enzymes have long been a source of inspiration for the development of efficient catalysts. Inspired by ferredoxin, Liu et al. designed Fe₂ DACs (N₂─Fe─N₂─Fe─N₂) using benzene‐1,2‐dithiol and CO‐protected Fe₂ dimers via the ZIF‐8 host‐guest encapsulation strategy for ORR (**Figure** **13**a‐d). The strategic addition of extra Fe and S sites near the main Fe sites serves as dual modulators for electronic structure adjustment. The resulting Fe₂N₆‐S catalyst exhibits improved ORR activity with an ultrahigh half‐wave potential of 0.921 V (vs RHE) and exceptional performance in Zn‐air batteries compared to single Fe site catalysts (Figure 13b,c). The introduction of Fe_2_ dual‐sites alters the O₂ adsorption configuration, activating the O─O bond, and facilitates the release of OH* through the downward shift of the *d*‐band center. Meanwhile, heteroatomic doping with S further alters the electronic structure, which accelerates the rate‐determining step involving OOH* formation (Figure 13a,d).^[^ ^127^ ^]^ Continuing with the theme of bio‐inspired catalysts, drawing from cytochrome *c* oxidases, Fe₂ nanozymes with direct Fe─Fe bonds (≈2.21 Å) were developed. These nanozymes display exceptional oxidase‐like activity (18.7 U mg⁻¹). The balanced O₂ adsorption/desorption capabilities of the Fe─Fe configuration enhance the antifouling performance of bioinspired membranes by 44.5‐fold. Additionally, this unique biomimetic mechanism utilizes electron‐proton pair transfers during enzyme‐like reactions, achieving a record osmotic energy conversion efficiency of 6.7 W m⁻^2^. These findings highlight the potential of Fe₂ nanozymes for advanced catalytic and energy applications.^[^ ^162^ ^]^

##### CO₂RR

Shifting the focus from ORR to CO₂RR, Fe₂ DACs with N₃─Fe─Fe─N₃ motifs have been developed for efficient CO₂ to CO reduction. It attains a FE_CO_ of 96.0% at −0.6 V (vs RHE) and a Tafel slope of 60 mV dec⁻¹, greatly outperforming single‐atom Fe catalysts. DFT calculations indicate that the Fe─Fe pairs facilitate CO₂ activation by bonding to its C and O atoms simultaneously, while the dual Fe centers reduce the energy barrier for CO desorption.^[^ ^9^ ^]^ Furthermore, another Fe₂ DACs shows a FE_CO_ above 80%, a turnover frequency of 26 637 h⁻¹, and superior durability compared to SACs (Figure 13e–h). Some research revealed that the orbital coupling between Fe_2_ sites decreases the energy gap for *CO adsorption (Figure 13e–h). A more advanced Fe‐Fe DACs with N₂─Fe─N₂─Fe─N₂ motifs was later reported, demonstrating superior activity compared to the N₃─Fe─Fe─N₃ system. The higher activity is ascribed to reduced CO poisoning on the Fe–Fe pairs in the N₂─Fe─N₂─Fe─N₂ motif, enhancing its catalytic efficiency.^[^ ^10^ ^]^ Moreover, utilizing the edge N atoms in the C₂N cavity as pincers, two neighboring Cu active sites were captured to construct N₂─Cu₂─N₂ active motifs with a strong Cu─Cu interaction of 2.42 Å. The adjacent Cu atom sites “flip” to be located on the same side of the C_2_N during the reaction to promote a synergistic effect for enhanced CO adsorption and dimerization, achieving a remarkable 71.7 % FE_ethylene_ at an applied potential of −1.1 V (vs RHE).^[^ ^128^ ^]^

##### N₂ORR

Expanding the scope beyond Fe‐based catalysts, using DFT calculations, a Fe₂ dual site catalyst anchored on N‐doped porous graphene (NG) was identified as the most active and selective for the novel 8e N₂ORR, enabling simultaneous N₂O removal and NH₃ synthesis (Figure 13i–l). The Fe‐Fe pairs adopt an N₃─Fe─Fe─N₃ configuration with a Fe─Fe bond length of 2.25 Å. This modulated structure promotes a bent N₂O adsorption configuration, facilitating hydrogenation via an 8e pathway to produce NH₃ (Figure 13j). The fabricated Fe₂/NG DACs achieve a FE of 77.8% and a high NH₃ yield of 2.9 mg h⁻¹ cm⁻^2^ at −0.6 V versus RHE in a neutral electrolyte (Figure 13k,l).^[^ ^129^ ^]^

##### Hydrogenation Reaction

In addition to the Fe‐based systems, highly dispersed Pt₂ clusters supported on mpg‐C₃N₄ were developed through a “precursor‐preselected” wet‐chemistry approach, using (ethylenediamine)iodoplatinum(II) dimer as the metal precursors. The mpg‐C₃N₄ substrate offers abundant anchoring N‐sites, stabilizing the metal species during the pyrolysis process. This process also removes organic ligands and prevents Pt₂ cluster agglomeration. The Pt_2_ pairs exhibit excellent catalytic performance for the highly selective hydrogenation of nitrobenzene to aniline, benefiting from the easy H₂ dissociation induced and the easy desorption of the aniline product.^[^ ^130^ ^]^ Likewise, Yun et al. developed a precise dynamic strategy for constructing atomically dispersed Pd₂ catalysts. Using Pd₂(F₃Ph₃P)₂(F₂PhS)₄ nanoclusters dispersed on an Al₂O₃ support (Figure 13m,n), they applied heat treatment in an H₂ atmosphere at 300 °C. During this process, ligand dissociation and new bond formation cause the in situ migration of Pd atoms, resulting in a Pd─Pd bond with a controllable interatomic distance of 2.7 Å. The synergistic effect of the Pd–Pd pairs contributes to high activity and selectivity for the semi‐hydrogenation of alkynes and functional internal acetylene (Figure 13m,n). This dynamic strategy highlights the potential to precisely control the formation of dual‐sites, leading to highly efficient and stable catalysts.^[^ ^163^ ^]^

#### 3.3.2 Heteronuclear Metal–Metal Pairs

Heteronuclear metal‐metal pairs have garnered significant research attention due to their ability to incorporate heterometallic atoms, offering enhanced control over the asymmetric electronic structure of the pairs. This asymmetry facilitates the selective activation of small‐molecule substrates at the active sites, enabling more efficient catalytic processes. With advancements in synthesis techniques, heteronuclear metal‐metal pair catalysts have become increasingly important, finding broad applications in HER, OER, CO₂RR, NO_3_RR, and urea oxidation.

##### HER

The ALD method effectively synthesizes heteronuclear metal pairs, exemplified by the two‐step ALD strategy that produced heteronuclear Pt‐Ni oxo‐dimers on C₃N₄ at 200 °C. Zhang et al. synthesized Pt─Ru pairs by selectively depositing Ru onto Pt/NCNTs (N‐doped carbon nanotubes). Since Ru atoms did not effectively attach to NCNTs during the initial ALD cycles, this enabled selective deposition of Ru onto Pt atoms, resulting in dimer structures comprising ≈70% of the material. The resulting Pt─Ru active motifs exhibited a Pt─Ru distance of 2.4 ± 0.4 Å, which could be easily changed from metal to semiconductor by the adsorption, leaving unoccupied orbitals. The interaction between H and Ru could be modulated by the Pt through the synergetic effect, which results in a mass activity of 23.1 A mg^−1^ at the overpotential of 0.05 V, which is 54‐fold greater than the Pt/C catalysts (0.43 A mg^−1^).^[^ ^131^ ^]^ Zhao et al. proposed a “navigation and positioning” strategy for precise and scalable photochemical synthesis of a series of heteronuclear dual‐sites (Zn−Ru, Ni−Ru, Zn−Cu, Co−Cu, Ni−Cu, and Bi−Cu) on PCN. Upon photoexcitation, negative charge accumulation occurs at the M_1_ nucleation site in M_A_/PCN, which can attach the secondary metal ion of M_B_ around the M_A_ site, constructing a series of heteronuclear DACs of M_A_−M_B_/PCN with M_A_−M_B_ distance of ≈2.5 Å. Take Zn−Ru/PCN DAC as an example, the proportion of dual‐site active motifs can reach 81%. In the photocatalytic hydrogen evolution, it was shown that the Ru site reduces H^+^ to H* and the Zn site acts as the H* desorption site, thus synergistically boosting activity.^[^ ^132^ ^]^ These studies highlight a promising and universal strategy for precise and scaled‐up synthesis of heteronuclear DACs by the irradiation of photosensitizer‐supported SACs.^[^ ^132^ ^]^

##### Hydrogenation and Dehydrogenation Reactions

**Figure 14:**
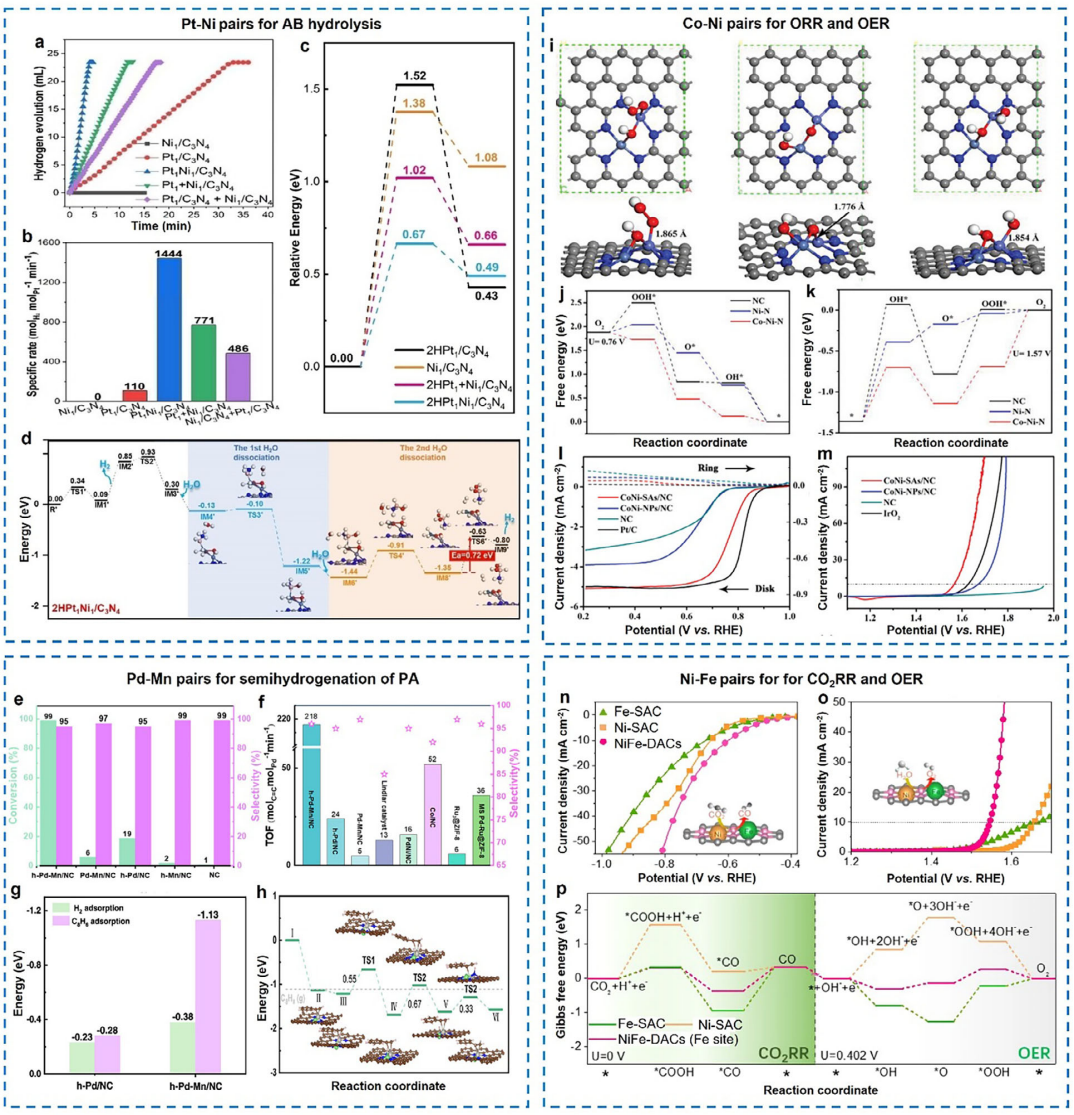
Heteronuclear metal‐metal pairs. a) Volume of hydrogen evolution versus reaction time for AB hydrolysis catalyzed by Ni_1_/C_3_N_4_, Pt_1_/C_3_N_4_, Pt_1_Ni_1_/C_3_N_4_, Pt_1_+Ni_1_/C_3_N_4_ and the physical mixture of Ni_1_/C_3_N_4_+Pt_1_/C_3_N_4_ at 25 °C. b) Specific rates of hydrogen evolution based on the Pt loadings. c) Energy diagrams of water dissociation over 2 HPt_1_/C_3_N_4_, Ni_1_/C_3_N_4_, 2 HPt_1_+Ni_1_/C_3_N_4_ and 2 HPt_1_Ni_1_/C_3_N_4_ catalysts (Due to the high reducibility of the AB molecule and strong H_2_ adsorption on Pt_1_, coordinated OH groups on Pt_1_ are expected to be replaced by two atomic hydrogens. Thus, the real active sites are 2 HPt_1_/C_3_N_4_, 2 HPt_1_+Ni_1_/C_3_N_4_, and 2 HPt_1_Ni_1_/C_3_N_4_). d) Calculated energy diagrams with important elementary steps for hydrolysis of AB on 2 HPt_1_/C_3_N_4_ catalysts. a–d) Reproduced with permission.^[^ ^133^ ^]^ Copyright 2022, Wiley‐VCH GmbH. e) Conversion and selectivity over h‐Pd‐Mn/NC, Pd‐Mn/NC, h‐Pd/NC, h‐Mn/NC, and NC. Reaction conditions: 5.0 mmol phenylacetylene, catalyst (7.5 mg of h‐Pd‐Mn/NC), 333 K, 1.5 MPa, 6 h, 10 mL *n*‐hexane. f) Comparison of TOF values over various catalysts for the semihydrogenation of phenylacetylene. g) Calculated H_2_ and C_8_H_6_ (phenylacetylene) adsorption energies over h‐Pd‐Mn/NC and h‐Pd/NC. h) Energy diagram of the semihydrogenation of phenylacetylene over h‐Pd‐Mn/NC and the side view of the corresponding intermediate structures. e–h) Reproduced with permission. Copyright 2024, American Chemical Society. i) Optimized atomic configurations of oxygen intermediates (OOH*, O*, and OH*) adsorbed on Co─Ni─N models. Free energy diagram of j) ORR, and k) OER processes on NC, Ni─N, and Co─Ni─N. l) ORR polarization curves at 1600 rpm of CoNi‐SAs/NC, CoNi‐NPs/NC, NC, and Pt/C. m) OER polarization curves at 1.62 V. i–m) Reproduced with permission. Copyright 2019. WILEY‐VCH Verlag GmbH & Co. KGaA, Weinheim. n) LSV curves of Fe‐SAC, Ni‐SAC, and NiFe‐DACs acquired on a rotating disc electrode at a rotation speed of 1600 rpm and a scan rate of 5 mV s^−1^. Catalyst loading is 0.1 mg cm^−2^ and electrolyte is CO_2_‐saturated 0.5 m KHCO_3_ solution. o) LSV curves for OER in O_2_‐saturated 1.0 m KOH at a rotation speed of 1600 rpm and a scan rate of 1 mV s^−1^. p) Calculated free energy diagrams for CO_2_RR and OER processes. n–p) Reproduced with permission.^[^ ^137^ ^]^ Copyright 2021, The Author(s), published by Nature Publishing Group.

The ALD method is effective in the preparation of heteronuclear metal pairs, exemplified by the two‐step ALD strategy that produced heteronuclear Pt‐Ni oxo‐dimers on C₃N₄ at 200 °C. Using MeCpPtMe₃ and nickelocene (NiCp₂) as precursors, N₂─Ni─O₂─Pt─O₂ motifs with an interatomic distance of 2.2 Å were confirmed by HAADF‐STEM and EXAFS. This catalyst demonstrated high performance in ammonia borane (AB) dehydrogenation, generating 23.4 mL of H₂ in 4 min, yielding an extremely high specific rate of 1444 mol_H2_ mol_Pt_^−1^ min^−1^, ≈13‐fold higher than that of the Pt_1_/C_3_N_4_ catalyst (**Figure** **14**a,b). Mechanistic studies revealed that the metals’ cooperative synergy enhances hydrogen evolution by improving water dissociation and facilitating B─H bond cleavage via a bridging OH group (Figure 14c,d).^[^ ^133^ ^]^ Liu et al. designed hollow mesoporous DACs via a host‐guest strategy by high‐temperature pyrolysis Pd(acac)₂ and Mn(acac)_3_ encapsulated in ZIF‐8 cages. These Pd─Mn pairs possessed superior thermodynamic stability, which facilitated the formation of Pd─Mn active motifs in DACs with a Pd─Mn bond of 2.3 Å. The introduction of atomically dispersed Mn with weak electronegativity drives electron transfer to Pd, regulating its electronic structure. Strong electronic coupling in Pd─Mn pairs enhanced the *d*‐electron domination near the Fermi level and promoted the adsorption of phenylacetylene and H_2_ on Pd active sites, thereby reducing the energy barrier for the semihydrogenation of phenylacetylene, resulting in a turnover frequency 16‐fold that of commercial Lindlar catalyst (Figure 14e,f).^[^ ^134^ ^]^

##### ORR and OER

Cui et al. developed Fe‐Mn DACs on ultrathin 2D NC nanosheets via a molten salt‐assisted pyrolysis strategy. The Fe─Mn bond distance is ≈2.3 Å. The achieved catalyst exhibits remarkable bifunctional activities for ORR and OER. Control experiments and DFT calculations reveal that the catalytic activity arises from the cooperative effect of the Fe‐Mn DACs aiding *OOH dissociation.^[^ ^135^ ^]^ Additionally, Han et al. developed a novel catalyst featuring atomically dispersed Co─Ni sites embedded in N‐doped hollow carbon nanocubes. These Co‐Ni DACs exhibit N_3_─Co─Ni─N_3_ active motifs with a Co─Ni bond length of 2.22 Å. They demonstrate remarkable ORR performance, achieving a high onset potential of 0.88 V, a half‐wave potential of 0.76 V, and a limiting current density of 4.95 mA cm^−2^. For anodic OER, an overpotential of 340 mV is required to reach a current density of 10 mA cm^−2^ (Figure 14l,m). The synergistic interaction between the neighboring Co and Ni centers enhances adsorption/desorption characteristics and reduces reaction barriers, thus facilitating efficient reversible oxygen electrocatalysis (Figure 14i–k).^[^ ^136^ ^]^

Electrochemical synthesis of DACs is an emerging and promising strategy. This approach leverages advanced in situ characterization techniques, such as operando X‐ray absorption spectroscopy (XAS), to thoroughly investigate the synergistic interactions between neighboring dynamic active sites during the synthesis process. Bai et al. demonstrated that electrochemical activation of Co species dispersed on CN in Fe‐containing alkaline electrolyte leads to incidental Fe incorporation, forming a Co‐Fe DAC. The Co single‐atom pre‐catalyst was initially achieved and deposited on a glassy carbon electrode, which can be active to adsorb [Fe(OH)_4_]^−^ under positive potentials, resulting in the dimeric Co···Fe active motifs, as confirmed by operando XAS. Notably, the absorption of Fe ions is reversible, and a fast equilibrium is established between the sorption of Fe ions.^[^ ^164^ ^]^ They further extended this strategy to synthesize Co‐Fe and Co‐Ni DACs from the corresponding single‐atom precursors via electrochemical transformation, offering a versatile platform for studying the catalytic mechanisms of heterogeneous OER electrocatalysts.^[^ ^165^ ^]^ It should be noted that some metal components can be oxidized to higher valence states during in situ activation. Although the stability of the DACs obtained through in situ electrochemical preparation requires further improvement, the above studies demonstrate that the controlled preparation of this class of DACs is highly feasible and promising.

##### CO₂RR and OER

Zeng et al. prepared a Ni─Fe catalyst via co‐pyrolyzing *L*‐alanine, Fe(CH₃COO)₂, Ni(CH₃COO)₂, and melamine in an Ar atmosphere. The Ni─Fe distance in the catalyst is ≈0.24 nm. Ni‐Fe DACs display extraordinary and stable electrocatalytic performance for CO_2_‐to‐CO conversion (50.4 mA cm^−2^ at an overpotential of 690 mV, FE of 94.5%) and O_2_ evolution (10 mA cm^−2^ at an overpotential of 310 mV) (Figure 14n,o). Mechanistic studies indicate the Ni─Fe pair contributes by altering orbital energy levels, creating unique electronic states, increasing the oxidation state of Fe, and weakening intermediate binding (Figure 14p).^[^ ^137^ ^]^

##### Urea Oxidation

Zhao et al. demonstrated that diethylenetriamine penta‐acetic acid acts as an effective precursor for stable bimetallic complexes through mild pyrolysis (≈250 °C), preventing surface migration. The long‐chain ligands with multiple COO─/N groups maintain fixed distances between metal centers, enabling precise synthesis of various metal‐metal pairs (e.g., Fe, Co, Ni, Mn) in carbon dots. Notably, the Ni–Mn pairs exhibit superior urea oxidation activity, achieving a low potential of 1.32 V at 10 mA cm^−2^, a Tafel slope of 40.1 mV s^−1^, and TOF and MA values of 0.98 s^−1^ (1.4 V) and 1155.92 A g_CDs_^−1^ (1.45 V), respectively.^[^ ^166^ ^]^

##### NO_3_^−^RR

Zhang et al. proposed Fe‐Co DACs where Fe^3^⁺ and Cu^2^⁺ are directly anchored to holey edge sites of NG, forming a N₂─Fe─Cu─N₂ motif with the Fe─Cu bonding distance of 2.3 Å. The catalyst enables an FE_NH3_ of 92.51% (at −0.3 V vs RHE) and a high NH_3_ yield rate of 1.08 mmol h^−1^ mg^−1^ (at −0.5 V vs RHE). Mechanistic studies show a relatively strong interaction between NO_3_^−^ and Fe‐Cu promotes the adsorption and discharge of NO_3_^−^ anions. N−O bonds are also shown to be weakened due to the existence of hetero‐atomic dual‐sites, which lowers the overall reaction barriers.^[^ ^138^ ^]^

## 4 Conclusions and Perspectives

Multifunctional catalysts featuring adjacent active motifs have emerged as foundational elements in heterogeneous catalysis, driving significant advancements across energy, environmental, and commercial sectors. By leveraging atomically close active motifs, these catalysts benefit from synergistic interactions and diverse catalytic systems, resulting in marked improvements in catalytic efficiency, selectivity, and stability. In this Review, we summarize various combinations of active motif pairs, including metal‐acid, metal‐base, and metal‐metal interactions, which exemplify synergistic cooperation beneficial for heterogeneous catalysis. Mechanisms such as double activation catalysis, cascade catalysis, and synergistic catalysis are explored, alongside the roles of active motifs and their corresponding structure‐activity relationships. These approaches ensure optimal proximity and interaction between motifs, laying a solid foundation for the development of multifunctional catalysts.

Characterizing the electronic and geometric structures of these catalysts is essential for understanding their structure‐reactivity relationships. Elucidating the structures of multifunctional catalysts with closely spaced active motifs requires a combination of techniques tailored to the materials involved. For amorphous supports, such as graphene oxide and amorphous carbon, electron microscopy and EXAFS are two core tools for revealing microenvironments. The absence of long‐range order complicates the use of conventional powder diffraction for crystallographic analysis. Conversely, for crystalline supports like zeolites and MOFs, high‐resolution SXRD, combined with Rietveld refinement and probe‐assisted protocols, provides crucial atomic‐level insights. Other spectroscopic techniques, including solid‐state NMR and electron paramagnetic resonance, can offer complementary information.

Looking ahead, we anticipate that a combination of in situ and *operando* characterization techniques will further offer extensive elucidation of structure‐reactivity relationships, which is key for developing more sophisticated and rational catalytic systems. While multifunctional catalysts have demonstrated impressive performance, key challenges remain: (i) precisely modulating the inter‐site distances between adjacent active motifs and (ii) accurately controlling their electronic properties. Despite numerous studies proposing various “optimized” catalytic systems, a universal solution for specific catalytic reactions remains elusive.

We believe that advancements in big data and machine learning will substantially enhance the development of this field. Artificial intelligence (AI) and machine learning are poised to revolutionize catalyst discovery by leveraging extensive datasets of material properties and literature reaction outcomes. These technologies are, and becoming increasingly, useful in predicting promising catalysts, optimizing synthesis parameters, and accelerating the screening of candidate materials. High‐throughput experimentation combined with AI‐driven analytics should greatly encourage researchers to identify trends and design rules at an unprecedented pace. Open‐access databases and collaborative research platforms will be crucial in fostering global innovation and ensuring reproducibility.

From an application perspective, there is growing potential for these multifunctional catalysts in emerging areas such as plastic upcycling, CO_2_ utilization, and nitrogen fixation. These challenging reactions often require the concerted action of multiple active sites, making them ideal testbeds for the concepts discussed in this review. Researchers should particularly focus on designing catalyst systems that can maintain their structural integrity and synergistic effects under harsh reaction conditions.

Future efforts should prioritize scalable and eco‐friendly synthesis strategies for heterogeneous catalysts with neighboring active motifs. While SACs have demonstrated scalable production, DACs require innovative approaches—such as modular assembly or defect engineering—to achieve precise spatial control and stability. Industrial‐scale challenges, including cost‐effective precursor utilization, reactor design for uniform active‐site distribution, and minimizing environmental footprints, must be systematically addressed. Integrating machine learning‐guided optimization with advanced characterization will accelerate the transition from lab‐scale breakthroughs to industrially viable cooperative catalysts.

By addressing these challenges, the field of heterogeneous catalysis can unlock its full potential, delivering innovative solutions to global energy, environmental, and sustainable development challenges. Combining cutting‐edge research with interdisciplinary collaboration will facilitate the design of next‐generation catalysts that are both efficient and environmentally responsible.

## Conflict of Interest

The authors declare no conflict of interest.
